# Association of clinical signs of possible serious bacterial infections identified by community health workers with mortality of young infants in South Asia: a prospective, observational cohort study

**DOI:** 10.1016/j.eclinm.2025.103070

**Published:** 2025-01-18

**Authors:** Gary L. Darmstadt, Saifuddin Ahmed, Mohammad Shahidul Islam, Safa Abdalla, Shams El Arifeen, Melissa L. Arvay, Abdullah H. Baqui, Zulfiqar A. Bhutta, Anuradha Bose, Nicholas E. Connor, Belal Hossain, Rita Isaac, Arif Mahmud, Dipak K. Mitra, Luke C. Mullany, Imran Nisar, Kalpana Panigrahi, Pinaki Panigrahi, Qazi Sadeq-ur Rahman, Senjuti Saha, Sajid B. Soofi, Nardos Solomon, Mathuram Santosham, Stephanie J. Schrag, Shamim A. Qazi, Samir K. Saha

**Affiliations:** a Department of Pediatrics, Stanford University School of Medicine, Stanford, CA, USA; b Department of Population, Family and Reproductive Health, Johns Hopkins Bloomberg School of Public Health, Baltimore, MD, 21205, USA; c Child Health Research Foundation, Dhaka, Bangladesh; d International Centre for Diarrhoeal Disease Research Bangladesh (icddrb), Dhaka, Bangladesh; e Centers for Disease Control and Prevention, Atlanta, GA, USA; f Department of International Health, Johns Hopkins University Bloomberg School of Public Health, Baltimore, MD, USA; g Centre for Global Child Health, The Hospital for Sick Children, Toronto, Ontario, Canada; h Institute for Global Health and Development, The Aga Khan University, Karachi, Pakistan; i Christian Medical College and Hospital Vellore, Vellore, India; j Department of Clinical Research, London School of Hygiene & Tropical Medicine, London, UK; k Department of Pediatrics and Child Health, The Aga Khan University, Karachi, Pakistan; l AIPH University, Bhubaneswar, India; m Department of Pediatrics, Georgetown University Medical Center, Washington, DC, USA; n Retired, World Health Organization, Geneva, Switzerland

**Keywords:** Newborn, Mortality, Sepsis, Possible serious bacterial infection, Community health workers, Integrated management of childhood illness, Danger signs

## Abstract

**Background:**

The World Health Organization (WHO) has developed guidance for community health workers (CHWs) in identifying sick young infants based on clinical signs. We conducted a prospective, observational cohort study to characterise mortality risk of young infants based on their clinical signs.

**Methods:**

We conducted a population-based, prospective observational cohort study at five sites in Bangladesh (Sylhet, November 01, 2011–December 31, 2013), India (Vellore and Odisha, September 01, 2013–February 28, 2015), and Pakistan (Karachi, January 01, 2012–December 31, 2013; Matiari, March 01, 2012–December 31, 2013) to identify newborn infants who were followed-up by CHWs through 10 scheduled home visits over the first 60 completed days after birth to identify signs of possible serious bacterial infection (PSBI). We determined the frequency of signs and conducted Cox regression to investigate the association of signs with mortality risk within 7 days of identification of the signs.

**Findings:**

CHWs made 522,309 visits to assess 63,017 young infants and found ≥1 sign(s) of PSBI at 14,245 visits (2.7%), including 5.8% (5568 of 96,390) and 1.8% (6635 of 365,769) of visits of infants 0–<3 and 7–<60 days of age, respectively. Each of the seven signs of PSBI when found alone was associated with significantly (p < 0.0001) increased risk for mortality, which increased further if any other additional sign of PSBI was found concurrently. Over the young infant period (days 0–<60) CHW identification of no movement or movement only on stimulation was associated with the highest risk for mortality [adjusted hazard ratio (aHR) 73.0, 95% confidence interval (CI) 44.4–119.9] followed by poor feeding (aHR 31.9, 95% CI 24.1–42.3) and hypothermia (<35.5 °C) (aHR 31.4, 95% CI 23.5–41.9). Hypothermia had particularly high risk for mortality during days 7–<60 (HR 45.1, 95% CI 27.6–73.4).

**Interpretation:**

WHO reconsideration of hypothermia as a sign of critical illness is warranted. Implementation research is urgently needed to reduce infant mortality by ensuring immediate referrals and interventions for children identified early by CHWs with no movement or movement only on stimulation, hypothermia, or poor feeding, especially in resource-poor settings.

**Funding:**

10.13039/100000865Bill and Melinda Gates Foundation, New Venture Fund for Global Policy and Advocacy.

**Research in context**

**Evidence before this study**

We searched for evidence during July–December 2021 and September 2024 associating signs of PSBI identified by CHWs with risk for mortality of young infants, initially in PubMed using search terms “bacterial infection (Title/Abstract)” AND “mortality (Title/Abstract)” OR “severe illness (Title/Abstract)” AND “young infants,” with no date or language restrictions. Additional searches replaced “bacterial infection” with specific signs including “fast breathing (Title/Abstract)”, “convulsions (Title/Abstract)”, “chest indrawing (Title/Abstract)”, or “poor feeding (Title/Abstract),” and snowball searches of reference lists were conducted, yielding 108 papers for review, including a single-site study in Bangladesh which analysed associations of signs of serious illness identified in the first week after birth by CHWs with mortality, and the three-country AFRINEST study which evaluated associations of current PSBI signs identified by CHWs with mortality of young infants.

**Added value of this study**

This three-country study of the Aetiology of Neonatal Infections in South Asia (ANISA) complements the three-country African Neonatal Sepsis Trials (AFRINEST) study in sub-Saharan African, enabling insights that have global relevance and providing the first prospective evidence from population-based surveillance which utilises time-varying analysis to examine associations of clinical signs of illness with risk for mortality over the young infant period. Kaplan–Meier analysis showed that the cumulative probability of mortality was highest for young infants (days 0–<60) presenting with no movement or movement only on stimulation as a single sign found alone (77.8%) and rose to 96.4% when combined with one or more other signs; these values for mortality probability associated with hypothermia reached 65.5% (found alone) and 92.3% [found with one or more other sign(s)]. Time-to-event analysis with Cox regression showed that no movement or movement only on stimulation was associated with the highest risk for mortality over the entire young infant period (days 0–<60) [adjusted hazard ratio (aHR) 73.0, 95% confidence interval (CI) 44.4–119.9], and during days 7–<60, infants had particularly high hazard for mortality in association with hypothermia (aHR 45.1, 95% CI 27.6–73.4).

**Implications of all the available evidence**

Hypothermia should be reconsidered as a sign of critical illness. Implementation research is needed to reduce infant mortality by ensuring immediate referrals and interventions for children identified early by CHWs with no movement or movement only on stimulation, hypothermia, or poor feeding, especially in resource-poor settings. Convulsions requires further definitional standardisation and improved identification.

## Introduction

An estimated 47% of 4.9 million deaths globally in under-five year old children occurred in the neonatal period (2.3 million) in 2022, four-fifths in sub-Saharan Africa (46%) and Southern Asia (34%).^1^ A recent prospective multi-country maternal and neonatal study in Africa and Asia reported that the most common causes of neonatal deaths are perinatal asphyxia (40%), severe neonatal infections (35%) and preterm birth complications (19%).^2^

In 2012 a systematic review with meta-analysis estimated that there were 6.9 million cases of possible serious bacterial infection (PSBI), an incidence of 7.6%, and a case-fatality rate (CFR) of 9.8% in sub-Saharan Africa, South Asia and Latin America.^3^ In 2019 the estimated global neonatal sepsis incidence was 28 cases per 1000 live births with 17.6% CFR.^4^ A prospective study in six countries in 2010–2013 reported an overall incidence of PSBI of 12.9% in the first 6 weeks after birth, with a CFR of 14.0%.^5^ A more recent prospective, 12-country study during 2015–2018 estimated the incidence of clinically suspected sepsis as 166 per 1000 live births and laboratory-confirmed sepsis incidence as 47 per 1000 live births.^6^

Several clinical signs of neonatal sepsis which can be identified by health workers at the front lines of care, such as community health workers (CHWs), have been documented^7^, ^8^, ^9^, ^10^ and validated.^11^, ^12^, ^13^ Based on multi-country evidence, the World Health Organization (WHO) identified seven signs of PSBI indicating need for referral of sick young infants for hospital care in the 2014 Integrated Management of Childhood Illness (IMCI), including not able to feed at all or not feeding well, convulsions, severe chest indrawing, high body temperature (38 °C or above), low body temperature (less than 35.5 °C), movement only when stimulated or no movement at all, or fast breathing (60 breaths per minute or more).^10^^,^^14^ The outcome used in studies to define signs of PSBI for use in IMCI guidelines has been need for hospitalisation.

In 2015, a WHO guideline^15^ recommended treatment of infants 7–59 days of age who present with isolated fast breathing with oral amoxicillin without referral. If other signs of PSBI are present and referral to a hospital is not feasible, the recommendation was also made to sub-classify PSBI and treat young infants on an outpatient basis with alternative regimens according to the severity of illness classification (Panel 1).^15^ Recent data from several countries where the current WHO guideline^16^ was implemented have shown that the majority of PSBI cases categorised as “clinical severe infection” and “severe pneumonia” were successfully treated on an outpatient basis with simplified antibiotic regimens, resulting in low mortality rates.^17^, ^18^, ^19^, ^20^, ^21^, ^22^, ^23^, ^24^, ^25^, ^26^, ^27^, ^28^

Panel 1 **Definitions and treatment for possible serious bacterial infection (PSBI) according to sub-classifications16**

PSBI is defined as young infants 0–59 days of age with any of the following signs: fast breathing (respiratory rate ≥60 breaths/minute in infants 0–6 days of age), severe chest in-drawing, high body temperature ≥38 °C, low body temperature <35.5 °C, no movement at all or movement only on stimulation, not able to feed at all or not feeding well and convulsions.^17^ *Sub-classifications and treatment*

• Pneumonia: Fast breathing (respiratory rate ≥60 per minute) as the only sign in infants 7–59 days old.
  o Recommended treatment: Oral amoxicillin twice daily for 7 days (given by a physician, nurse or clinical officer working at a health facility).
• Severe pneumonia: Fast breathing (respiratory rate ≥60 per minute) as the only sign in infants 0–6 days old.
  o Recommended treatment: Referral to the hospital. If referral is not feasible, treat with oral amoxicillin twice daily for 7 days.
• Clinical severe infection: Not feeding well, movement only on stimulation, severe chest indrawing, high body temperature ≥38 °C, or low body temperature <35.5 °C
  o Recommended treatment: Referral to the hospital. If referral is not feasible, treat with injections of gentamicin once daily for 2 days or 7 days plus oral amoxicillin twice daily for 7 days.
• Critical Illness: Presence of any of the following signs – convulsions, not able to feed at all, or no movement on stimulation.
  o Recommended treatment: Refer URGENTLY to hospital; if referral is still not possible, treat with daily injectable gentamicin and twice-daily injectable ampicillin until referral is possible or for at least 7 days.

The revised young infant IMCI algorithm^16^ based on the 2015 WHO guideline^15^ currently gives equal importance to all the PSBI signs for referral, except for fast breathing in infants 7–59 days of age, for which outpatient treatment with oral antibiotics by a physician, nurse or clinical officer working at a health facility is recommended. However, recent studies which have examined associations of signs of PSBI with mortality have found that the CFR associated with individual signs may vary—as implied by sub-categorisation into pneumonia (fast breathing in infants 7–59 days of age), severe pneumonia (fast breathing in the first week after birth), clinical severe infection (i.e., not feeding well, movement only on stimulation, severe chest indrawing, fever, or hypothermia), and critical illness (i.e., presence of convulsions, not able to feed at all, or no movement on stimulation) (Panel 1)—and presence of single or multiple signs may be associated with differing risks for mortality of young infants with PSBI.^5^^,^^13^^,^^29^^,^^30^ Implementation of management of PSBI would be aided by identification of sick young infants with low risk of mortality, based on their clinical signs, who can be treated on an outpatient basis, thereby reducing the number of young infants needing referral for treatment in a hospital. Furthermore, use of mortality as the outcome in assessing risk associated with signs of PSBI, rather than need for hospitalisation, may enhance policy relevance.

We conducted an analysis of data from the Aetiology of Neonatal Infection in South Asia (ANISA) study^31^ to inform further distinction of risk for mortality of young infants based on their clinical signs and to further inform management recommendations on whether infants with particular signs need urgent referral to a hospital or could more readily be treated on an outpatient basis. In this paper we take a perspective grounded in IMCI and analyse the prevalence of clinical signs and the mortality risk of young infants associated with signs of PSBI identified by CHWs. In a companion paper, we validate CHW identification of signs in comparison with physician assessment and examine risk of mortality in association with signs of PSBI identified during physician assessment.

## Methods

### Study sites and procedures

**Fig. 1:**
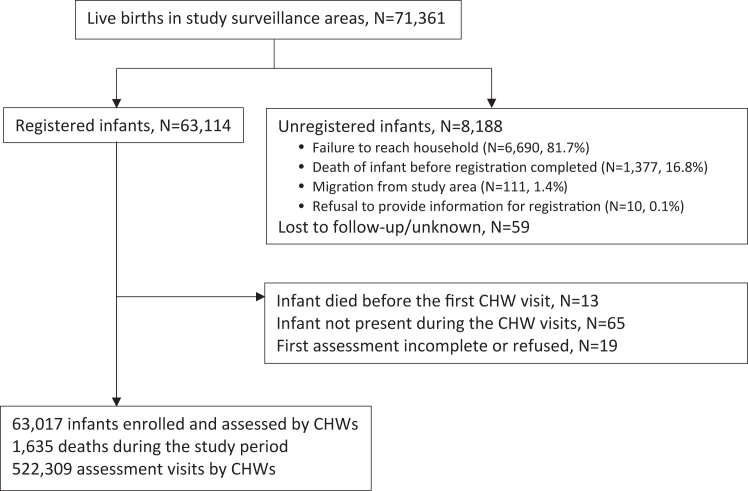
**Study flow diagram for assessment of young infants for signs of possible serious bacterial infection by community health workers in the Aetiology of Neonatal Infection in South Asia (ANISA) study**.

The ANISA study was a prospective, observational cohort study conducted in five sites in Bangladesh (Sylhet, November 1, 2011–December 31, 2013), India (Vellore and Odisha, September 1, 2013–February 28, 2015), and Pakistan (Karachi, January 1, 2012–December 31, 2013; Matiari, March 1, 2012–December 31, 2013).^31^^,^^32^ Population-based demographic surveillance included registering married women of reproductive age (13–49 years) in the target communities, identifying pregnancies, identifying live births as soon as possible after delivery, identifying cases of PSBI among young infants in the community, and referring cases of suspected infection to study hospitals. Unmarried women were excluded, given the remote possibility of a birth to an unmarried woman in the study sites. Experts from WHO trained supervisory staff from the study sites, who in turn trained CHWs from their respective sites for 15–21 days.^32^ Infants who were born in the study site surveillance areas and were visited by CHWs and assessed for signs of PSBI within 7 days of birth were enrolled; there were no exclusion criteria for purposes of followup for assessment of signs of PSBI (Fig. 1).

### CHW assessments for signs of PSBI

Enrolled infants were followed-up by CHWs through the first 60 days after birth (the young infant period) during up to ten scheduled home visits, ideally on day 0 (24 h), day 2 (72 h), day 6 (end of the first week) and then every seven days. Additionally, CHWs also visited and assessed infants when informed by families that their baby was sick. In each visit, CHWs assessed infants for signs of PSBI as defined by WHO (Table 1) and recorded their findings using a standarised case record form.^16^^,^^32^

**Table 1:** Clinical signs of possible serious bacterial infection as defined and assessed by community health workers (CHWs) and physicians during the Aetiology of Neonatal Infection in South Asia study.

Clinical signs	Definition followed	Assessment procedures	
		CHW assessment	Physician assessment
Fast breathing	Respiratory rate **≥**60 breaths per minute	Used acute respiratory infection (ARI) timer to count the number of times the chest raised in 60 s	Used ARI timer to count the number of times the chest raised in 60 s
Severe chest indrawing	Inward movement of the lower chest wall when the child breathes in	Observed the lower chest wall and whether it went IN when the baby breathed IN	Observed the lower chest wall and whether it went IN when the baby breathed IN
Fever	Temperature ≥38 °C	Recorded axillary temperature using a digital thermometer	Recorded axillary temperature using a digital thermometer
Hypothermia	Temperature <35.5 °C	Recorded axillary temperature using a digital thermometer	Recorded axillary temperature using a digital thermometer
No movement or movement only on stimulation	Decreased child capacity for spontaneous movement of the body	Observed whether the baby moved limb or eye only on tactile stimulation	Observed whether the baby moved limb or eye only on tactile stimulation
Convulsions	Stiffening of the arms and legs because of muscles contraction	Parental statement or observed convulsion. Used local terms like ‘fits’ or ‘spasm.’	Parental statement or observed convulsion. Used local terms like ‘fits’ or ‘spasm.’
Poor feeding	Child is not able to feed at all or stopped feeding well	Parental statement and observed whether the infant was positioned properly, latched and sucked effectively when breastfeeding, after position and attachment were adjusted if required.	Parental statement and observed whether the infant was positioned properly, latched and sucked effectively when breastfeeding, after position and attachment were adjusted if required. If observation was not possible, the tip of the clean fifth finger was used to assess the sucking reflex.

CHWs referred PSBI cases to study physicians at a health care facility—only indicating need for referral, not information on their assessment for signs of PSBI—where physicians independently identified signs of PSBI (Table 1) and made a clinical diagnosis of suspected infection, which prompted evaluation for infection as reported previously.^31^^,^^32^^,^^33^ Vital status of infants was assessed by CHWs at each visit, and verbal autopsy was performed for young infant deaths in the study sites using standard WHO verbal autopsy methods.^34^, ^35^, ^36^, ^37^, ^38^, ^39^ Details of study implementation at each of the sites have been published previously.^40^, ^41^, ^42^, ^43^, ^44^ Infants were managed clinically according to usual care of the health facilities at the study sites; CHWs did not provide treatments.

### Analysis

All visits (scheduled and family-initiated care-seeking) where the infant was present and assessed by a CHW were included in the analysis. We carried out descriptive analysis of the frequency of visits where each of the seven signs of PSBI was found alone, as well as where signs were found in combinations. We followed STROBE guidelines for cohort studies, taking into account the recommendation of the Strengthening the Reporting of Observational Studies in Epidemiology for Newborn Infection,^45^ and stratified these analyses by the following time frames: 0–<3 days, 3–<7 days, 0–<7 days, and 7–<60 days.

We conducted time-to-event analysis with Cox hazards regression models to investigate the association of finding a given sign on assessment of young infants by CHWs—from the first visit to the end of 60 days after birth, during days 0–<60 when up to 10 scheduled visits plus sick-child visits occurred—with hazard for all-cause mortality compared to not finding that sign. We applied a time dependent definition for the exposure and conducted time-varying hazard analysis to address immortality bias within the survival analysis framework as the literature suggests that the time-dependent Cox model consistently provides unbiased estimates.^46^, ^47^, ^48^ Any infant who was never visited or who died before a CHW visit was left censored,^49^ and exposure periods of unknown status pertaining to the presence of signs of PSBI before the first visit were eliminated, ensuring that the correct exposure period reflecting at-risk for mortality outcome was assessed. Kaplan–Meier probabilities of mortality were calculated to display the results over the young infant period.^50^ We defined the period of risk for mortality as extending for 7 days after finding a sign of PSBI; when death occurred more than 7 days after identification of the clinical sign with no subsequent CHW visit information, mortality was not attributed to that sign. Sensitivity analysis was conducted using a period of risk that extended 14 days after identification of the sign of PSBI. We used Cox regression analysis to examine three different patterns of CHW identification of each sign (a single sign found alone, the sign found with at least one other sign, any sign(s) found other than the particular sign) compared to not finding any sign in association with all-cause mortality and displayed Kaplan–Meier probabilities of mortality over the young infant period (first visit to the end of the 60th day, days 0–<60). Cox regression was used to also examine the association between the presence of clinical signs and the occurrence of mortality during time frames of the first CHW visit to the end of day 3 (days 0–<3), the first visit to the end of day 7 (days 0–<7), and day 7 to the end of the 60th day (days 0–<60); if the death occurred outside the time frame under consideration, then the death was not attributed to the sign(s) identified during that time period. Thus, if a sign was identified on day 5, with death on day 8, the infant would be at risk of death from the identification of the sign to day 7 in the analysis of the 0–<7 day period, but the death would be not considered an outcome in this analysis (censored at day 7 before death occurred). For analysis of the 7–<60 day period, the period of risk would include days 7 and 8, and for the 0–<60 day analysis, the infant would at risk on days 5–8, and the death would be included in analyses of both the 7–<60 and the 0–<60 day periods. Analyses were adjusted for maternal education, presence of pregnancy complications, presence of labour complications, place of birth (home vs facility), and preterm birth. We did not have detailed information on how infants were managed in the hospital or on the time interval between CHW/physician assessment and commencement of treatment, and thus did not take these factors into account in our analysis. The analysis was conducted with SAS 9.4 and Stata 18.5. An alpha cut-off of 0.05 was used to determine statistical significance.

### Ethical approval

For the parent ANISA study, informed verbal consent was obtained from pregnant women when they were registered in the study. The study was approved by the ethics committees or internal review boards of all participating organisations. The analysis of data from the ANISA study was approved by the Stanford Institutional Review Board (protocol # 55341).

### Role of the funding source

The funders of the study had no role in study design, data collection, data analysis, data interpretation, or writing of the report. S. Ahmed, S. Abdalla, and G.L.D. had full access to the data in the study and take responsibility for the integrity and accuracy of the data analysis; G.L.D. had responsibility for the decision to submit the manuscript for publication.

## Results

### Study sample

There were 71,361 live births among eligible women in the five study sites during the study period; 63,114 infants were registered in the ANISA study, 8188 were unregistered, and 59 were lost to follow-up (Fig. 1, Table 2). The two primary reasons for lack of registration of infants whose families resided in the study sites were that they could not be reached by a CHW (6690 of 8118, 81.7%) or the infant was found to have died before registration (1377 of 8118, 16.8%) (Fig. 1). Maternal and household characteristics were similar for infants who were registered in the ANISA study compared to those who were not registered (Table 2). The preterm birth rate was higher for unregistered (32.0%, 2171 of 6778) than registered (19.8%, 11,836 of 59,877) infants, and is reflected in the higher mortality rate [168.4 per 1000 infants, 95% confidence interval (CI) 160.4–176.7] among unregistered infants (Table 2). Mothers of infants who were registered were a median age of 27 years [interquartile range (IQR) 23–31], 57.7% (36,157 of 62,631) had ever attended school, 39.4% (24,505 of 62,203) received ≥4 antenatal care visits, 45.5% (28,700 of 63,052) gave birth at home, 80.5% (50,387 of 62,631) owned a mobile telephone, and for 32% (20,189 of 63,114) of mothers the infant was firstborn. Registered infants were a median age of 11.6 h (IQR 4.3–24.9) at first contact and 51.4% were male (32,419 of 63,114), 27.3% were low birthweight (16,832 of 61,569), and the mortality rate before the end of the 60th day was 26.7 (95% CI 25.5–28.0] per 1000 live births.

**Table 2:** Maternal, household and infant characteristics of livebirths in the Aetiology of Neonatal Infections in South Asia (ANISA) study.

Characteristic (% unless otherwise specified)^a^	Enrolled (N = 63,114)	Unregistered (N = 8188)	Total (N = 71,302)^b^
**Maternal**			
Age at delivery (median years, IQR^c^) (n = 71,298)	27 (23–31)	27 (23–31)	27 (23–31)
Ever attended school/madrasha (n = 70,527)	36,157 (57.7)	4526 (57.3)	40,683 (57.7)
Poor nutritional status^d^ (n = 71,302)	6267 (10.0)	696 (8.5)	6.963 (9.8)
Received full antenatal package^e^ (n = 70,183)	24,505 (39.4)	2975 (37.3)	27,480 (39.2)
At least one antenatal care visit with a skilled provider (n = 70,183)	48,984 (78.8)	6004 (75.2)	54,988 (78.4)
Birth location (n = 71,206)			
Health facility	34,351 (54.4)	5037 (61.5)	39,388 (55.2)
Home	28,700 (45.5)	5037 (61.5)	31,817 (44.6)
Skilled birth attendant^f^ (n = 71,302)	35,140 (55.7)	5198 (63.5)	40,338 (56.6)
Clean delivery kit (n = 71,294)	37,530 (59.5)	4735 (57.8)	42,265 (59.3)
First birth (n = 71,298)	20,189 (32.0)	2901 (35.4)	23,090 (32.4)
Presence of pregnancy complications	19,126 (30.0)	2809 (34.3)	22,926 (31.1)
Presence of labour complications	10,365 (16.4)	2061 (25.2)	13,371 (18.2)
**Household**			
Household members [median (IQR)] (n = 70,527)	6 (4–9)	6 (4–10)	6 (4–9)
Electricity (n = 70,527)	48,817 (77.9)	6508 (82.4)	55,325 (78.5)
Piped water (n = 70,527)	19,735 (31.5)	2667 (33.8)	22,402 (31.8)
Mobile phone ownership (n = 70,527)	50,387 (80.5)	6281 (79.6)	56,668 (80.4)
**Infants**			
Age at registration (median hour, IQR) (n = 63,114)	11.6 (4.3–24.9)		11.6 (4.3–24.9)
Sex (n = 71,290)			
Boys	32,419 (51.4)	4277 (52.3)	36,696 (51.5)
Girls	30,695 (48.6)	3899 (47.7)	34,594 (48.5)
Preterm (n = 66,655)	11,836 (19.8)	2171 (32.0)	14,007 (21.0)
Low birthweight (n = 61,569)	16,832 (27.3)	–	16,832 (27.3)
Deaths (n = 71,302)	1689	1378	3067
Mortality among infants <60 days per 1000 livebirths^g^ (95% confidence interval)	26.7 (25.5–28.0)	168.4 (160.4–176.7)	43.0 (41.5–44.5)

a Sample sizes are shown in parentheses; differences between sample sizes and 71,302 are missing values.

b 59 infants were lost to follow-up and are not included.

c IQR: Interquartile range.

d Defined as mid-upper-arm circumference <21.5 cm.

e Receipt of at least four antenatal visits from a community health worker or skilled healthcare providers.

f Qualified doctor, nurse, midwife, or paramedic.

g Mortality of the unregistered babies includes deaths within 7 days of birth.

Following delivery, 13 infants died before the first visit, 65 infants could not be found when the household was reached after birth, and 19 assessments could not be completed (Fig. 1). A total of 63,017 infants were consented and enrolled in the study. CHWs made 522,309 home visits (scheduled and family-initiated) to assess these 63,017 infants between birth and 60 completed days; 77% of infants (N = 48,415) had eight or more scheduled visits. One or more signs of PSBI were identified by CHWs during 14,245 (2.7%) visits (Fig. 1, Table 3).

**Table 3:** Frequency of signs of possible serious bacterial infection (PSBI)^a^ found by community health workers during home visits among 63,017 infants, shown by infant age in days.

Time frame (days)	Total number of visits	Any sign	Fast breathing	Chest indrawing	Fever	Hypothermia	No movement	Convulsions	Poor feeding
		n (%)	n (%)	n (%)	n (%)	n (%)	n (%)	n (%)	n (%)
0–<3	96,390	5568 (5.8%)	3089 (3.2%)	410 (0.4%)	1029 (1.1%)	1045 (1.1%)	491 (0.5%)	325 (0.3%)	1532 (1.6%)
3–<7	60,148	2041 (3.4%)	1206 (2.0%)	145 (0.2%)	411 (0.7%)	221 (0.4%)	161 (0.3%)	129 (0.2%)	396 (0.7%)
7–<60	365,771	6636 (1.8%)	4091 (1.1%)	1636 (0.4%)	949 (0.3%)	321 (0.1%)	389 (0.1%)	381 (0.1%)	1155 (0.3%)
0–<60	522,309	14,245 (2.7%)	8386 (1.6%)	2191 (0.4%)	2389 (0.5%)	1587 (0.3%)	1041 (0.2%)	835 (0.2%)	3083 (0.6%)

a Fast breathing (=respiratory rate ≥60 breaths per minute), chest indrawing (=severe chest indrawing), fever (=temperature ≥38 °C), hypothermia (=temperature <35.5 °C), no movement (=no movement or movement only on stimulation), convulsions (by maternal report), poor feeding (=not able to feed at all or stopped feeding well).

### Signs of PSBI identified by CHWs during home visits

Any one or more of the signs of PSBI was found in 5.8% (5568 of 96,390) of visits of infants 0–<3 days of age, and in 3.4% (2041 of 60,148) and 1.8% (6636 of 365,771) of visits of infants 3–<7 and 7–<60 days of age, respectively (Table 3). The frequency of finding each of the signs was highest during visits of infants 0–<3 days and was lowest for infants 7–<60 days, except for severe chest indrawing which had similar frequencies (0.4% of visits; 0–<3 days: 410 of 96,360, 7–<60 days: 1635 of 365,771) in both age groups.

**Fig. 2:**
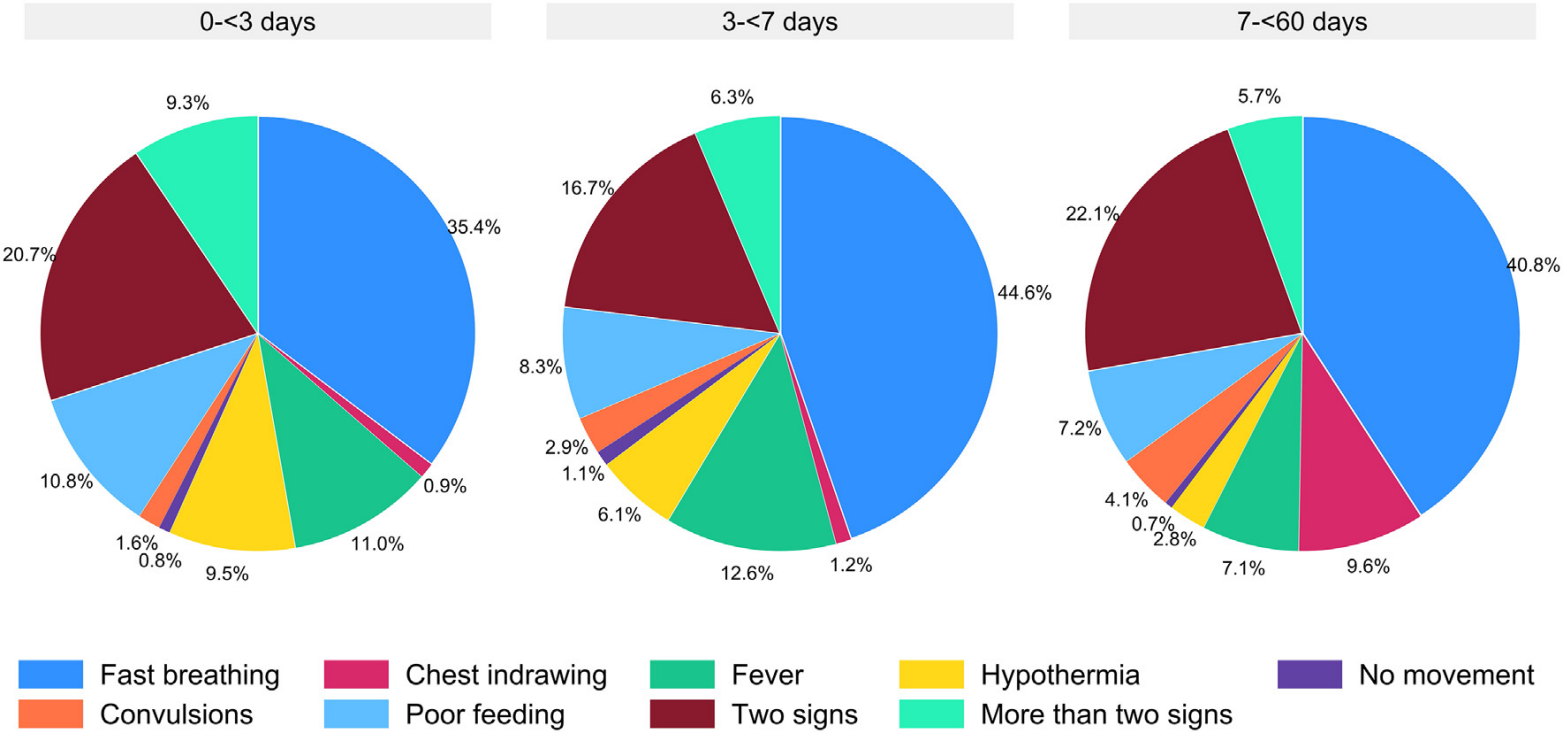
**Distribution of signs of possible serious bacterial infection (PSBI)**^**a**^**found singly, in pairs and with more than two signs, considering community health worker visits of young infants ages 0–<60 days in which one or more sign of PSBI was found, by age groups**. ^**a**^Fast breathing (=respiratory rate ≥60 breaths per minute), chest indrawing (=severe chest indrawing), fever (=temperature ≥38 °C), hypothermia (=temperature <35.5 °C), no movement (=no movement or movement only on stimulation), convulsions (by maternal report), poor feeding (=not able to feed at all or stopped feeding well).

Considering the presence of the seven signs relative to one another in infants 0–<3 days of age, the sign most commonly found alone was fast breathing [35.4% (1971 of 5568 visits) of cases where one or more sign was found] followed by fever [11.0% (611 of 5568)] and poor feeding [10.8% (604 of 5568)] (Fig. 2). Similarly in infants 3–<7 days of age, fast breathing was found most commonly alone [(44.6% (911 of 2041)], followed by fever [12.6% (258 of 2041)] and poor feeding [8.3% (169 of 2041)]. Severe chest indrawing, no movement or movement only on stimulation, and convulsions were the least common single signs (1–3%) in these age groups. In infants 7–<60 days of age, fast breathing [41.0% (2657 of 6483 visits)] was also the sign most commonly found alone, followed by severe chest indrawing [9.3% (605 of 6483)], poor feeding [7.3% (475 of 6483)] and fever [6.9% (449 of 6483)]. In the three age groups, two signs were found concurrently in about one-fifth (17–22%) of visits where a sign was found by the CHW. Further information about overlap between signs can be found in Supplemental Tables S1–S3. Notably, in 73.3% (407 of 555) of instances where severe chest indrawing was found, fast breathing was also identified in infants 0–<7 days; conversely, however, fast breathing was accompanied by severe chest indrawing in only 9.5% (407 of 4295) of cases (Supplemental Table S2); the pattern was similar in infants 0–<3 days of age (Supplemental Table S1). In infants 7–<60 days of age, just over half (56.0%, 917 of 1636) of those with severe chest indrawing also had fast breathing (Supplemental Table S3) and about one-fifth (22.4%, 917 of 4091) of infants who had fast breathing also had severe chest indrawing. Nearly three-fourths (70–74%) of infants across all age groups who had no movement or movement only on stimulation also had poor feeding (Supplemental Tables S1–S3).

### Association of signs of PSBI identified by CHWs with mortality

#### Young infant period (0–<60 days)

**Fig. 3:**
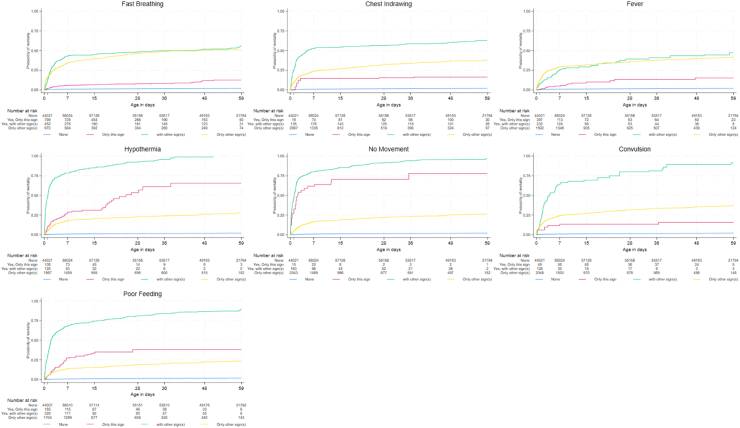
**Kaplan–Meier probabilities for mortality of young infants ages 0-<60 days by signs of possible serious bacterial infection**^**a,b**^**identified by community health workers**. ^a^Cox regression analysis was used to examine patterns of CHW identification of each sign: a single sign found alone (only this sign), the sign found with at least one other sign (with other signs), any sign(s) found other than the particular sign (only other sign(s)) compared to not finding any sign (none) in association with all-cause mortality. ^**b**^Fast breathing (=respiratory rate ≥60 breaths per minute), chest indrawing (=severe chest indrawing), fever (=temperature ≥38 °C), hypothermia (=temperature <35.5 °C), no movement (=no movement or movement only on stimulation), convulsions (by maternal report), poor feeding (=not able to feed at all or stopped feeding well).

Kaplan–Meier probabilities for mortality were analysed for each sign of PSBI as a binary variable (the sign was present or not during days 0–<60) (Supplemental Figure S1) and by four categories: when found alone, when accompanied by one or more other signs, when the sign was not found but one or more other signs were found, or when no sign was found (Fig. 3, Supplemental Table S4). The cumulative probability of mortality was highest for young infants presenting with no movement or movement only on stimulation as a single sign found alone (77.8%) and rose to 96.4% when combined with one or more other signs; for hypothermia, these figures for mortality probability reached 65.5% (hypothermia found alone) and 92.3% [hypothermia found with one or more other sign(s)]. Infants identified with poor feeding as a single sign showed moderate probability of mortality (38.1%) over the young infant period; when another sign or signs appeared along with poor feeding, the probability of mortality was heightened to 89.3%. Fast breathing, severe chest indrawing, fever, and convulsions as single signs had similar patterns as one another and lower levels of probability for mortality (12–16%) compared to no movement or movement only on stimulation, hypothermia, and poor feeding, although the risk associated with convulsions was particularly heightened in association with another sign, reaching 91.6% probability of mortality. Noteably, the probability of mortality was similar for fast breathing found with one or more other signs compared to only other signs without fast breathing, suggesting that fast breathing did not add to the risk for mortality when other signs of PSBI were present; a similar pattern was found for fever.

All signs of PSBI when found singly were significantly (p < 0.0001) associated with hazard for mortality in young infants during days 0–<60 in unadjusted and adjusted Cox regression analyses (Table 4). Cox regression showed stronger associations with mortality for all the signs when the sign was present with ≥1 other sign than when the sign was found alone. No movement or movement only on stimulation (HR 73.0, 95% CI 44.4–119.9) had the highest risk for mortality followed by poor feeding (HR 31.9, 95% CI 24.1–42.3) and hypothermia (HR 31.4, 95% CI 23.5–41.9). For each of these signs, hazard for mortality was higher when the sign was found alone compared to when only other sign(s) were found. In contrast, the adjusted hazard ratio for mortality associated with fast breathing, severe chest indrawing, fever, or convulsions when found alone was lower, in the range of 5–7, and the hazard was lower than when only other sign(s) were found. Furthermore, for fever, the hazard for mortality was similar to higher when only other signs were found (aHR 25.9, 95% CI 23.2–28.8) than when fever was found along with one or more other sign (aHR 23.4, 95% CI 17.7–31.0), suggesting that fever contributed little additional risk. Results were similar for sensitivity analyses examining associations of signs with mortality within 14 days.

**Table 4:** Association of community health worker (CHW) identification of signs of possible serious bacterial infection (PBSI)^a^ alone or in combination with other signs of PBSI with mortality of infants 0–<60 days, (522,309 CHW assessments, 63,017 infants, 1635 deaths).

Sign^a^	Category^b^	CHW visits (N)	Infants (N)	Total exposure time (days)	Deaths (N)	Mortality rate/1000 child-days	Hazard ratio (HR)						
							Unadjusted			Adjusted^c^			
							HR	95% confidence limits		HR	95% confidence limits		p-value
Fast breathing	Yes (only this sign)	5580	4039	18,696	58	3.1	5.9	4.5	7.7	5.5	4.2	7.2	<0.0001
	Yes (with other sign/s)	2783	2448	9479	212	22.4	46.1	39.7	53.6	40.4	34.7	47.0	<0.0001
	No (but other sign/s)	5829	4940	19,163	370	19.3	36.6	32.4	41.4	31.9	28.2	36.1	<0.0001
	No (no sign)	508,117	62,411	3,070,686	995	0.3	1.0						
Chest indrawing	Yes (only this sign)	707	614	4491	6	1.3	5.5	2.5	12.4	5.0	2.2	11.2	<0.0001
	Yes (with other sign/s)	1478	1269	6553	110	16.8	47.5	39.0	57.8	40.5	33.2	49.4	<0.0001
	No (but other sign/s)	12,007	9067	36,294	524	14.4	24.7	22.1	27.6	22.3	19.9	24.9	<0.0001
	No (no sign)	508,117	62,411	3,070,686	995	0.3	1.0						
Fever	Yes (only this sign)	1338	1243	4356	20	4.6	6.7	4.3	10.4	5.8	3.7	9.1	<0.0001
	Yes (with other sign/s)	1045	999	3331	53	15.9	26.3	19.9	34.8	23.4	17.7	31.0	<0.0001
	No (but other sign/s)	11,809	8605	39,651	567	14.3	28.8	25.9	32.0	25.9	23.2	28.8	<0.0001
	No (no sign)	508,117	62,411	3,070,686	995	0.3	1.0						
Hypothermia	Yes (only this sign)	838	715	1772	50	28.2	41.5	31.2	55.3	31.4	23.5	41.9	<0.0001
	Yes (with other sign/s)	735	631	1302	194	149.0	181.8	154.5.	213.8	126.2	106.5	149.5	<0.0001
	No (but other sign/s)	12,619	9333	44,264	396	8.9	18.3	16.2	20.6	17.0	15.1	19.2	<0.0001
	No (no sign)	508,117	62,411	3,070,686	995	0.3	1.0						
No movement	Yes (only this sign)	113	106	328	16	48.8	84.6	51.6	138.9	73.0	44.4	119.9	<0.0001
	Yes (with other sign/s)	914	816	2310	243	105.2	166.1	143.5	192.2	136.5	117.5	158.6	<0.0001
	No (but other sign/s)	9684	9684	44670	381	8.5	16.9	15.0	19.1	15.3	13.6	17.3	<0.0001
	No (no sign)	508,117	62,411	3,070,686	995	0.3	1.0						
Convulsions	Yes (only this sign)	415	346	2008	6	2.9	7.8	3.5	17.4	6.7	3.0	15.0	<0.0001
	Yes (with other sign/s)	415	375	977	81	82.9	99.1	78.5	125.0	84.7	67.1	107.1	<0.0001
	No (but other sign/s)	13,362	9869	44,352	553	12.5	24.3	21.8	27.1	21.8	19.6	24.4	<0.0001
	No (no sign)	508,117	62,411	3,070,686	995	0.3	1.0						
Poor feeding	Yes (only this sign)	1.246	1152	3070	53	17.3	28.9	21.8	38.2	31.9	24.1	42.3	<0.0001
	Yes (with other sign/s)	1809	1610	4340	321	73.9	117.4	102.8	134.0	101.4	88.5	116.2	<0.0001
	No (but other sign/s)	11,113	8136	39,906	265	6.6	13.7	11.9	15.7	12.2	10.6	14.0	<0.0001
	No (no sign)	508,005	62409	3,070,206	994	0.3	1.0						

a Fast breathing (=respiratory rate ≥60 breaths per minute), chest indrawing (=severe chest indrawing), fever (=temperature ≥38 °C), hypothermia (=temperature <35.5 °C), no movement (=no movement or movement only on stimulation), convulsions (by maternal report), poor feeding (=not able to feed at all or stopped feeding well).

b Cox regression analysis was used to examine patterns of CHW identification of each sign: a single sign found alone (only this sign), the sign found with at least one other sign (with other signs), any sign(s) found other than the particular sign (only other sign(s)) compared to not finding any sign in association with all-cause mortality.

c Adjusted for maternal education, place of birth, history of labor and pregnancy complications, preterm birth.

#### First 3 days (0–<3 days)

During the first 3 days (0–<3 days), patterns of mortality risk were similar as for the young infant period (0–<60 days). All signs found singly during the first 3 days were significantly (p < 0.0001) associated with hazard for mortality, and the hazard of mortality increased further for all the signs when one of more additional sign(s) were found concurrently (Table 5). Infants identified by CHWs to have no movement or movement only on stimulation had the highest hazard for mortality in the first 3 days (HR 100.5, 95% CI 51.2–197.2), followed by hypothermia (HR 30.2, 95% CI 19.2–57.5), severe chest indrawing (HR 27.4, 95% CI 8.7–85.8) and poor feeding (HR 26.3, 95% CI 17.3–40.3). For no movement or movement only on stimulation, hypothermia, and poor feeding as single signs, but not for severe chest indrawing, hazard for mortality was higher than when only other signs were found, consistent with the particularly high risk conferred by these three signs. Risk for mortality was lower in association with convulsions (HR 11.6, 95% CI 3.7–36.1), fast breathing (HR 6.9, 95% CI 4.7–10.3) and fever (HR 5.1, 95% CI 2.6–10.0). The hazard for mortality for convulsions or for fast breathing was markedly increased when other signs were also present (HR 84.9 and 62.2, respectively) to levels greater than when signs other than convulsions or fast breathing were present (HR 30.0 and 40.2, respectively), suggesting that convulsions and fast breathing contributed to the mortality risk in association with other signs. In contrast, the hazard for mortality for fever found with one or more other signs (HR 20.6) was lower compared to when only other signs without fast breathing was found (HR 39.6), suggesting that fever did not add to the risk for mortality when other signs of PSBI were present.

**Table 5:** Association of community health worker (CHW) identification of signs of possible serious bacterial infection (PSBI)^a^ alone or in combination with other signs of PSBI with mortality of infants 0–<3 days, (151,543 CHW assessments, 56,899 infants, 593 deaths).

Sign^a^	Category^b^	CHW visits (N)	Infants (N)	Total exposure time (days)	Deaths (N)	Mortality rate/1000 child-days	Hazard ratio (HR)						
							Unadjusted			Adjusted^c^			
							HR	95% confidence limits		HR	95% confidence limits		p-value
Fast breathing	Yes (only this sign)	2683	2228	2141	28	13.0	7.2	4.9	10.7	6.9	4.7	10.3	<0.0001
	Yes (with other sign/s)	1371	1279	1032	140	135.6	71.3	56.7	88.1	62.2	50.2	77.2	<0.0001
	No (but other sign/s)	3196	2850	2402	199	82.8	45.2	37.3	54.7	40.2	33.1	48.9	<0.0001
	No (no sign)	144,293	56,092	128,586	226	1.8	1.0						
Chest indrawing	Yes (only this sign)	81	79	61	3	49.4	29.7	9.5	92.6	27.4	8.7	85.8	<0.0001
	Yes (with other sign/s)	476	447	347	63	181.7	96.2	77.7	127.3	82.1	61.8	109.0	<0.0001
	No (but other sign/s)	6693	5.638	5168	301	58.2	31.7	26.6	37.6	28.9	24.2	34.5	<0.0001
	No (no sign)	144,293	56,092	128,586	226	1.8	1.0						
Fever	Yes (only this sign)	818	772	881	9	10.2	5.6	2.9	11.0	5.1	2.6	10.0	<0.0001
	Yes (with other sign/s)	549	530	531	23	43.6	23.5	15.3	36.1	20.6	13.4	31.8	<0.0001
	No (but other sign/s)	5883	4903	4163	335	80.5	43.6	36.8	51.6	39.6	33.3	47.0	<0.0001
	No (no sign)	144,293	56,092	128,586	226	1.8	1.0						
Hypothermia	Yes (only this sign)	644	586	314	21	66.9	36.9	23.6	57.7	30.2	19.2	57.5	<0.0001
	Yes (with other sign/s)	604	541	313	131	418.4	214.9	172.8	267.3	164.7	130.4	207.9	<0.0001
	No (but other sign/s)	6002	5058	4948	215	43.4	23.8	19.7	28.0	22.6	18.7	27.3	<0.0001
	No (no sign)	144,293	56,092	128,586	226	1.8	1.0						
No movement	Yes (only this sign)	65	64	42	9	216.6	124.0	63.7	241.4	100.5	51.2	197.2	<0.0001
	Yes (with other sign/s)	558	513	374	162	432.7	226.3	184.5	277.6	182.6	147.9	225.3	<0.0001
	No (but other sign/s)	6627	5556	5159	196	38.0	20.8	17.2	25.1	19.1	15.8	23.2	<0.0001
	No (no sign)	144,293	56,092	128,586	226	1.8	1.0						
Convulsions	Yes (only this sign)	135	125	129	3	23.2	13.2	4.2	41.2	11.6	3.7	36.1	<0.0001
	Yes (with other sign/s)	296	272	257	51	198.4	103.3	76.1	140.1	84.9	62.2	115.8	<0.0001
	No (but other sign/s)	6819	5723	5189	313	60.3	32.8	27.7	39.0	30.0	25.2	35.7	<0.0001
	No (no sign)	144,293	56,092	128,586	226	1.8	1.0						
Poor feeding	Yes (only this sign)	746	695	490	24	49.0	26.4	17.3	40.2	26.3	17.3	40.3	<0.0001
	Yes (with other sign/s)	1123	1017	744	210	282.2	147.7	122.1	178.6	123.6	1010.6	150.3	<0.0001
	No (but other sign/s)	5359	4538	4329	132	30.5	16.9	13.6	20.9	15.3	12.3	19.1	<0.0001
	No (no sign)	144,244	56,082	128,544	226	1.8	1.0						

a Fast breathing (=respiratory rate ≥60 breaths per minute), chest indrawing (=severe chest indrawing), fever (=temperature ≥38 °C), hypothermia (=temperature <35.5 °C), no movement (=no movement or movement only on stimulation), convulsions (by maternal report), poor feeding (=not able to feed at all or stopped feeding well).

b Cox regression analysis was used to examine patterns of CHW identification of each sign: a single sign found alone (only this sign), the sign found with at least one other sign (with other signs), any sign(s) found other than the particular sign (only other sign(s)) compared to not finding any sign in association with all-cause mortality.

c Adjusted for maternal education, place of birth, history of labor and pregnancy complications, preterm birth.

#### First week (0–<7 days)

Results for infants in the first 7 days (Supplemental Table S5) were similar to those for the first 3 days, although risk of mortality associated with poor feeding (HR 37.0, 95% CI 27.0–50.7) was higher than for severe chest indrawing (HR 17.6, 95% CI 6.6–47.1). The lower hazard for mortality for fever in combination with other signs (HR 25.4) compared to when other signs besides fever were found (HR 36.0) suggests that fever added little to the risk for mortality.

#### Young infant period after the first week (7–<60 days)

In infants 7–<60 days of age, severe chest indrawing when found alone was not significantly associated with hazard for mortality; the other six signs of PSBI were significantly associated with risk for mortality in this time period (Table 6). Associations with mortality were significant and stronger when CHWs identified one or more additional signs in combination with each of the seven signs compared to when any one sign was found alone. No movement or movement only on stimulation (HR 62.7, 95% CI 23.4–168.2) and hypothermia (HR 45.1, 95% CI 27.6–73.4) had the highest hazards for mortality among signs found as singly, followed by poor feeding (HR 22.5, 95% CI 11.6–43.6). Risk for mortality was lower for fast breathing (HR 4.2, 95% CI 2.6–6.9), convulsions (HR 4.3, 95% CI 1.1–17.4), and fever (HR 10.4, 95% CI 5.2–21.0). Hazard for mortality for fast breathing found with one or more other signs (HR 16.4) was lower compared to when other signs without fast breathing was found (HR 26.1), suggesting that fast breathing did not add to the risk for mortality when other signs of PSBI were present.

**Table 6:** Association of community health worker (CHW) identification of signs of possible serious bacterial infection (PSBI)^a^ alone or in combination with other signs of PSBI with mortality of infants 7–<60 days (365,773 CHW assessments, 59,704 infants, 768 deaths).

Sign^a^	Category^b^	CHW visits (N)	Infants (N)	Total exposure time (days)	Deaths (N)	Mortality rate/1000 child-days	Hazard ratio (HR)						
							Unadjusted			Adjusted^c^			
							HR	95% confidence limits		HR	95% confidence limits		p-value
Fast breathing	Yes (only this sign)	2698	2046	13,931	17	1.2	4.6	2.8	7.5	4.2	2.6	6.9	<0.0001
	Yes (with other sign/s)	1371	1193	7598	33	4.3	18.2	12.8	25.8	16.4	11.6	11.6	<0.0001
	No (but other sign/s)	2515	2220	14,428	98	6.8	28.2	22.8	34.9	26.1	21.1	21.1	<0.0001
	No (no sign)	359,189	59,569	2,714,592	620	0.2	1.0						
Severe chest indrawing	Yes (only this sign)	634	550	4265	2	0.4	2.1	0.5	8.6	1.9	0.9	7.8	0.35
	Yes (with other sign/s)	996	843	5766	27	4.6	20.0	13.6	29.4	17.4	11.8	25.6	<0.0001
	No (but other sign/s)	4954	3958	25,926	119	4.6	18.0	14.7	21.9	16.8	13.8	20.5	<0.0001
	No (no sign)	359,189	59,569	2,714,592	620	0.2	1.0						
Fever	Yes (only this sign)	469	451	2894	8	2.8	11.5	5.7	23.2	10.4	5.2	21.0	<0.0001
	Yes (with other sign/s)	474	461	2401	15	6.2	25.6	15.3	42.7	26.3	15.7	44.0	<0.0001
	No (but other sign/s)	5641	4288	30,662	125	4.1	16.3	13.5	19.8	15.0	12.3	28.2	<0.0001
	No (no sign)	359,189	59,569	2,714,592	620	0.2	1.0						
Hypothermia	Yes (only this sign)	182	144	1055	17	16.1	58.2	35.8	94.5	45.1	27.6	73.4	<0.0001
	Yes (with other sign/s)	126	110	731	40	54.7	195.8	141.5	271.1	133.3	95.5	186.0	<0.0001
	No (but other sign/s)	6276	4862	34,170	91	2.6	10.9	8.7	13.6	10.3	8.2	12.8	<0.0001
	No (no sign)	359,189	59,569	2,714,592	620	0.2	1.0						
No movement	Yes (only this sign)	45	44	218	4	18.4	65.5	24.5	175.4	62.7	23.4	168.2	<0.0001
	Yes (with other sign/s)	330	306	1563	52	33.3	132.7	99.7	176.4	121.3	90.8	167.0	<0.0001
	No (but other sign/s)	6209	4779	34,176	92	2.7	10.9	13.6	13.6	10.0	8.1	12.5	<0.0001
	No (no sign)	359,189	59,569	2,714,592	620	0.2	1.0						
Convulsions	Yes (only this sign)	268	229	1683	2	1.2	5.1	1.3	20.2	4.3	1.1	17.4	0.038
	Yes (with other sign/s)	108	101	578	13	22.5	89.5	51.6	155.1	77.0	44.4	133.7	<0.0001
	No (but other sign/s)	6208	4802	33,695	133	3.9	15.9	13.1	19.2	14.7	12.1	17.7	<0.0001
	No (no sign)	359,189	59,569	2,714,592	620	0.2	1.0						
Poor feeding	Yes (only this sign)	473	452	2137	9	4.2	17.2	8.9	33.2	22.5	11.6	43.6	<0.0001
	Yes (with other sign/s)	654	614	2989	63	21.1	85.0	65.5	110.4	83.7	64.2	109.0	<0.0001
	No (but other sign/s)	5455	4149	30,824	76	2.5	10.0	7.8	12.6	8.9	7.0	11.4	<0.0001
	No (no sign)	359,134	59,565	2,714,212	619	0.2	1.0						

a Fast breathing (=respiratory rate ≥60 breaths per minute), chest indrawing (=severe chest indrawing), fever (=temperature ≥38 °C), hypothermia (=temperature <35.5 °C), no movement (=no movement or movement only on stimulation), convulsions (by maternal report), poor feeding (=not able to feed at all or stopped feeding well).

b Cox regression analysis was used to examine patterns of CHW identification of each sign: a single sign found alone (only this sign), the sign found with at least one other sign (with other signs), any sign(s) found other than the particular sign (only other sign(s)) compared to not finding any sign in association with all-cause mortality.

c Adjusted for maternal education, place of birth, history of labor and pregnancy complications, preterm birth.

## Discussion

Each of the seven signs of PSBI found individually by CHWs was associated with significant risk for mortality during the young infant period (days 0–<60), thus confirming the importance of their inclusion in the young infant IMCI protocol and signaling a need for active management of infants who present to CHWs with any of the signs of PSBI.^16^ Mortality in association with each sign identified by CHWs was further elevated when another sign or signs was also found, increasing the urgency for a referral. Across all age groups [0–<3 days, 0–<7 days, days 7–<60, and the entire young infant period (days 0–<60)], no movement or movement only on stimulation and hypothermia had the strongest independent associations with mortality while poor feeding was intermediate in risk in all age groups (although hazard for mortality over days 0–<60 was similar to that for hypothermia). Severe chest indrawing was intermediate in risk for mortality in the first 3 days after birth but was not associated with risk for mortality after the first week. Convulsions, fever and fast breathing had lower but consistently significant associations with mortality in all age groups, although evidence for fever as a risk factor in the first week and for fast breathing after the first week was realtively weak.

Infants who present with no movement or movement only on stimulation need particularly urgent management and referral to the hospital, given the 100-fold elevated hazard for mortality during the first 3 days and 73-fold hazard over the young infant period (days 0–<60) compared to when no sign was found. However, even in a health systems context in which CHWs were trained to refer all infants with signs of PSBI to health facilities for physician assessment and management, the cumulative Kaplan–Meier probability of mortality for infants presenting to CHWs with no movement or movement only on stimulation as a sole sign reached 78% over the young infant period, and 96% when combined with one or more other signs. Risk associated with hypothermia as a single sign was also ominous: 31-fold elevated hazard and 66% probability of mortality in the young infant period, increasing to 92% probability of mortality when combined with one of more other signs. Thus, for infants who present with these signs, greater urgency of intervention is required. Compared to hypothermia, poor feeding as a single sign showed similar to slightly less risk (32-fold hazard and 38% probability for mortality over the young infant period), but urgency is magnified when accompanied by one or more other signs of PSBI (89% risk of mortality in the young infant period). Signs pertaining to impaired movement, temperature regulation and feeding could reflect a moribund state—a final common pathway—arising in the context of a variety of conditions and disease states. We classified deaths of the infants in the study using standard verbal autopsy methodology and found similar distributions of causes of death as reported previously for other populations (unpublished data).^2^^,^^51^ However, the ability to precisely disentangle the specific causes of death in infants with these signs is beyond the diagnostic capabilities of verbal autopsy in settings such as ours where medical autopsy—such as is being done now in CHAMPS sites^52^—is unavailable.

Our study complements the African Neonatal Sepsis Trials (AFRINEST) in which CHWs also visited infants up to 10 times during the first two months after birth at five sites in three sub-Saharan African countries (the Democratic Republic of the Congo, Kenya and Nigeria).^29^^,^^30^^,^^53^^,^^54^ The ANISA and AFRINEST studies were of comparable size and were substantially similar in design concerning CHW assessment.^29^, ^30^, ^31^^,^^32^ In AFRINEST, CHW identification of signs of PSBI prompted referral of young infants to study nurses for further assessment and referral for enrollment in antibiotic treatment trials,^29^^,^^30^ whereas CHWs in the ANISA study referred young infants to physicians for assessment and management, including determination of aetiology of infection.^31^^,^^32^^,^^33^ In contrast, the six-country Global Network for Women and Children's Health Research study in Kenya, Zambia, Pakistan, India, Argentina and Guatemala utilised physician and nurse identification of some of the current signs of PSBI.^5^ For AFRINEST, Puri et al.^29^ reported CFR by signs of PSBI identified by CHWs in the community, whereas Nisar et al.^30^ reported CFR by signs of PSBI confirmed by nurses at health facilities. We followed a similar approach; here, we focused on household assessments by CHWs as the starting point for young infant IMCI. In a companion paper, we examine agreement between CHW and physicians assessments of signs and mortality risk in association with signs identified by physicians at health facilities.

Our analysis has important implications for sub-classification and treatment of infants identified with signs of PSBI by CHWs. Fast breathing was identified most frequently but was among the signs with the lowest risk for mortality, as also found in AFRINEST^29^^,^^30^ and the NICHD Global Network.^5^ Our data corroborate the current IMCI algorithm for outpatient treatment of fast breathing in young infants 7–59 days of age,^15^^,^^16^ which is further supported by low rates of mortality observed among young infants 7–<60 days of age with isolated fast breathing in Bangladesh, Ethiopia, India and Malawi who were successfully treated in the community by CHWs.^55^ Prior analysis of ANISA data using partial latent class Bayesian modeling showed that among young infants with late-onset isolated fast breathing, illness was attributed to any infectious aetiology in only 18% of cases and half of these were attributed to viral infection.^33^ Fever also carried a relatively low risk for mortality when identified as a single sign by CHWs in the first week, as was also found in the AFRINEST study.^29^ Our findings for convulsions contrast with the current WHO algorithm which includes convulsions as one of three single signs of critical illness, as we found relatively low risk for mortality associated with convulsions, especially after the first week (HR 4.3). Identification of convulsions by CHWs is based primarily on reporting by mothers, who may find it challenging to discern convulsions from other movements of newborn infants. When CHWs reported the presence of other signs of PSBI alongside convulsions, risk for mortality was markedly increased (e.g., HR 77 and 95 in combination with other signs during days 0–<7 and 7–<60, respectively). Thus while assessment for convulsions is important, urgent management is most salient when other signs accompany a report of convulsions. In the AFRINEST study using mothers’ reports of convulsions, CHWs found few infants with convulsions and no deaths,^29^ while nurses at health facilities found a relatively high CFR.^30^ Hibberd et al. found variable rates of mortality across sites (6–50%) among infants identified with convulsions by physicians and nurses in the first 6 weeks.^5^ Risk of mortality of young infants reported by CHWs to have convulsions requires further research, including greater standardisation in the definition and more reliable means of identifying the sign in young infants. The current IMCI algorithm also includes “not able to feed at all” and “no movement on stimulation” as signs of critical illness.^16^ The ANISA study used modified definitions of these signs: “not able to feed at all or stopped feeding well” and “no movement or movement only on stimulation,” respectively (Table 1).^14^^,^^32^ This study corroborates that young infants with these signs, particularly, no movement or movement only on stimulation, even when defined more broadly, have high risk for mortality. As in our study, the AFRINEST study also found the highest rate of mortality in the first 3 day to first week after birth when CHWs identified the sign of no movement or movement only on stimulation.^29^ ANISA data also showed that young infants with illness attributable to bacterial infection had significantly higher proportions of hypothermia, no movement or movement only on stimulation, convulsions, and poor feeding than infants with a viral aetiology.^33^ Moreover, among infants ages 0–<3 days, there was a significantly higher proportion of infants with no movement or movement only on stimulation and poor feeding in the bacterial than the viral aetiology group. Based on this analysis, it was suggested that hypothermia may be considered a sign of critical illness along with convulsions and movement only on stimulation.^33^ The current IMCI algorithm puts hypothermia as a sign of clinical severe infection instead of critical illness,^16^ whereas we found that hypothermia was a highly ominous sign throughout the young infant period. In the AFRINEST study, Nisar et al. also found that the mortality rate was highest for hypothermia (11.0%) among signs of clinical severe infection identified by CHWs and verified by nurses.^30^ They reported a similar mortality rate for convulsions (11.3%) and higher rates of mortality for unable to feed at all (22.9%) and no movement at all (25.0%), all three of which are categorised as signs of critical illness. Hibberd et al. also found the highest risk for mortality for hypothermia among the five single signs of PSBI that they evaluated.^5^ Moreover, epidemiologic research has demonstrated high risk of mortality associated with hypothermia.^56^ Taken together, these data suggest that reconsideration should be given to categorising hypothermia in young infant IMCI guidelines as a sign of critical illness, particularly given the exceeding high probability for mortality found in our analysis.

One or more signs of PSBI were found in nearly 6% of visits of infants 0–<3 days of age and nearly 2% of visits of infants 7–<60 days of age. Similarly, the AFRINEST study found one or more signs of PSBI in 2% of home visits.^29^ In about one-fifth of episodes of PSBI, two or more signs were found together and thus risk for mortality was heightened, similar to the prospective, multi-country NICHD Global Network study^5^ and the combined data from AFRINEST and the Simplified Antibiotics Treatment Trials conducted in Bangladesh and Pakistan.^57^ Ours is the first study to report associations among signs, finding that in nearly three-fourths (73%) and just over half (56%) of instances where severe chest indrawing was found, fast breathing was also identified in the first week after birth and during days 7–<60, respectively. This is important because while severe chest drawing as a single sign was not associated with risk for mortality during days 7–<60, the presence of another sign or signs alongside severe chest indrawing signalled a significantly increased mortality risk. Considering the converse, fast breathing was accompanied by severe chest indrawing in about one in ten infants in the first week (days 0–<7) and in about one-fifth of cases in the late young infant period (days 7–<60) (Supplemental Table S2). Fast breathing can be isolated and transient, particularly in the first days after birth due to delay in clearance of fetal lung fluid after birth, leading to ineffective gas exchange, respiratory distress, and tachypnoea.^58^ Nearly three-fourths of infants across all age groups who had poor feeding also had no movement or movement only on stimulation. On the other hand, about one-quarter of infants with no movement or movement only on stimulation also had poor feeding.

Our results suggest that earlier detection of illness and/or more rapid management of young infants with no movement or movement only on stimulation or hypothermia, even as single signs, beyond that currently provided through the health systems at our study sites, is required to reduce their risk for mortality, as also emphasised by Hibberd et al.^5^ This also applies for the finding of poor feeding, especially in combination with another sign of PSBI, which most commonly was fast breathing, no movement or movement only on stimulation, or hypothermia (in the first week) or fever (days 7–<60). The need for further refinement of treatment strategies based on the differential risk of mortality by signs of PSBI was also noted by AFRINEST.^30^ Public health strategies for improving child survival require that signs are detected early enough and prompt appropriate treatment is provided to prevent poor outcomes. Currently, this appears to not occur in situations in South Asia or sub-Saharan Africa when the system is dependent on CHW identification of these signs during routinely scheduled home visits, which may not happen regularly.^21^^,^^24^ Further research and programmatic efforts are urgently needed to avert deaths in young infants with no movement or movement only on stimulation and hypothermia, as well as poor feeding. Of note, a recent multi-country implementation research initiative of case management of young infants with PSBI when the referral was not possible achieved high overall coverage (76%) and low mortality rates of 1.9% for infants with clinical severe infection and 14.6% for infants with a critical illness (Panel 1).^59^ Thus, reducing mortality of young infants with PSBI—even with signs of critical illness—is possible through active identification and quality case management of illness.

While mothers have been shown to have limited capability to identify signs of PSBI,^60^ they can potentially play a critical role in the early identification of danger signs. Where CHW visits are of lower frequency and possibly lower quality, mothers may be best positioned to notice illnesses earlier than CHWs. This is supported by a PSBI implementation study in Palwal, India,^24^ where social mobilisation activities empowered communities, families and mothers to recognise sick young infants and seek appropriate care. However, in Ethiopia, while two-thirds of mothers knew one or more danger signs, only 11% had experienced a newborn danger sign.^61^ Further research is needed to determine how to equip caregivers in early danger sign recognition, and to improve health system performance to enable earlier detection of signs of PSBI and to limit the lag time between identification of signs and the initiation of appropriate therapy. This may be an important opportunity for the potential use of telehealth or call center technology to facilitate access to clinical assessment of infants in response to caregiver concerns.

Strengths of this study were its large size and inclusion of several representative sites from three South Asian countries. This study complements the AFRINEST study in three sub-Saharan African countries, and together, they enable us to draw insights that have global relevance. Despite its large size, analyses across seven signs of PSBI and three time periods resulted in small sample sizes at the level of stratification of mortality as our primary outcome. Rather than calculating CFRs, we modelled risk for mortality using rigorous Cox regression methods which take time-varying covariates into account. There was some limitation in comparing ANISA and AFRINEST results because ANISA grouped no movement with movement only on stimulation and not able to feed at all with stopped feeding well, whereas AFRINEST examined the clinical components of these signs together in the community but separately at health facilities.^29^^,^^30^ There may also have been variations in the assessments by the CHWs at our five different sites, although efforts were made to standardise their assessments.

Additional analyses of our data should examine differences by sex of the infants. Additional multivariable analyses which examine associations with various combinations of signs and their interactions would be informative. Due to the large number of combinations of signs to account for in such a multivariable analysis, sparsity-promoting machine learning methods (e.g., lasso or elastic net) may be an effective approach to identify the associations and combinations of signs most significantly linked with mortality. Future research could also examine associations of individual signs and combinations of signs of PSBI with mortality due to infections—expanding on the analyses presented by Arvay et al.^33^—as well as with mortality due to birth asphyxia, preterm birth and congenital malformations as identified by verbal autopsy^34^, ^35^, ^36^, ^37^, ^38^, ^39^ or possibly by medical autopsy.^52^ Such analyses could potentially expand the utility of algorithms such as IMCI to identify sick young infants and to rationalise the implementation of interventions, including antibiotic therapy, to save lives.

In conclusion, with the availability of evidence from ANISA (South Asia) in addition to AFRINEST (sub-Saharan Africa), global themes can now be drawn on the importance of signs of PSBI and their association with mortality of young infants. Although each of the signs was associated with risk for mortality, the implications for finding them varied, with no movement or movement only on stimulation, hypothermia, and poor feeding portending particularly high risk and urgency for intervention. Consideration should be given to classifying hypothermia as a critical sign in young infant IMCI guidelines. Further research on standardisation of the definition, consistency and reliability in recognition of convulsions is also warranted. Overall, our data support plans by WHO to oversee a randomised controlled trial in Africa and Asia to evaluate whether isolated fever or fast breathing in infants 0-<7 days of age—the signs with the lowest risk for mortality—can be treated on an outpatient basis compared to hospitalisation; the planned inclusion of infants in this age range with severe chest indrawing requires careful monitoring.^62^

## Contributors

G.L.D., S.Ahmed, S.Abdalla, S.A.Q., M.S.I. and S.K.S. conceptualised the paper; G.L.D. and N.S. led literature review; S.Abdalla, M.S.I., and S.Ahmed managed data curation; formal analysis was conducted by S.Ahmed, S.Abdalla, and M.S.I. S.Ahmed, S.Abdalla and M.S.I. verified the underlying data; investigation was conducted by site investigators M.S.I., S.E.A., A.H.B., Z.A.B., A.B., N.E.C., B.H., R.I., A.M., D.K.M., I.N., K.P., P.P., Q.S.R., S.S., S.B.S., M.S., S.K.S.; methodology was devised by G.L.D., S.Ahmed, and S.Abdalla; G.L.D. and S.K.S. acquired funding, managed project administration and resources, and provided supervision; G.L.D., S.A.Q., M.S.I. and S.K.S. validated study findings; G.L.D., S.Ahmed, and S.Abdalla led data visualisation; G.L.D. led writing of the original draft; S.A.Q. and M.S.I. provided critical input to paper drafts; all authors contributed to writing review and editing. All authors contributed intellectual content and approved the final draft for publication. S.Ahmed, S.Abdalla, M.S.I. and G.L.D. had full access to the data in the study and take responsibility for the integrity and accuracy of the data analysis; G.L.D., S.Ahmed, and M.S.I. had responsibility for the decision to submit the manuscript for publication.

## Data sharing statement

The data collected as part of the ANISA study were shared with all participating collaborators. Data will be shared outside the participating collaborators based on reasonable requests to SKS with scientific rational and sound methodology.

## Declaration of interests

The authors declare no conflicts of interest.

## Appendix A Supplementary data

ANISA_Data-Form-4A_version 6.0_06.06

ANISA_Data-Form-4B_FuV-7-59days_version 6.0_06.06

ANISA_Data-Form-6_P_version 6.0_06.06
