# Supplementary material for: Association of clinical signs of possible serious bacterial infections identified by community health workers with mortality of young infants in South Asia: a prospective, observational cohort study
Source: eClinicalMedicine. 2025 Jan 18;80:103070. doi: 10.1016/j.eclinm.2025.103070 (PMC11787667; doi:10.1016/j.eclinm.2025.103070)
Supplement: ANISA_Data-Form-4A_version 6.0_06.06 [file mmc1.pdf]

Form 4A: Newborn Routine Follow-up, 1st week

STUDY ID							
1	-					-	

4A CLUSTER CODE |___|___|___|

Scheduled Visit	
Day 2 ☐	Day 6☐

ANISA

This form is to be completed for all newborns enrolled in surveillance, for the scheduled visits in the first week after birth, (i,e. days 2 and 6). In case the newborn is not present on the scheduled visit date, one re-visit may be attempted

**1. Address and identification information**							
1.01	**Country/Site**		**BANGLADESH/ SYLHET**		**1**		
1.02	**Upazila**						
1.03	**Union**						
1.04	**Village**						
1.05	**Bari**						
1.06	**Household**						

1.11	Woman's Current ID					-				-			-		
1.12	Woman's Permanent ID					-				-			-		

1.13	Woman's Name	
1.14	Husband's Name	
1.15	Household Head's Name	
1.16	CHW's Name & Code	

1.21	Date of Visit (1st attempt)							
1.22	Date of Visit (2nd attempt)							
1.23	Date of Visit (3rd attempt)	d	d	m	m	y	y	y	y

1.24	Time of interveiw	Start time |__|__|: |__|__| (h h m m) End time |__|__|: |__|__| (h h m m)

Form 4A: Newborn Routine Follow-up, 1st week

STUDY ID							
1 -						-	

No	Questions	Responses	Skip
1.31	[***Visit Outcome***]	Completed .... 1<br>Incomplete .... 2<br>Refused follow-up .... 3<br>No-one present to respond after 2 visits..... 4<br>Out-migrated 5	 <br>→STOP<br>→STOP<br>→STOP
1.32	[***Respondent***]	Mother .... 1<br>Grand Mother .... 2<br>Aunt .... 3<br>Sister .... 4<br>Father .... 5<br>Grand father .... 6<br>Uncle .... 8<br>Brother .... 9<br>Other (Specify__________________........... 7	
1.41	[***Is the Mother alive?***]	Yes, Mother is alive .... 1<br>No, Mother has died 2	→1.51
1.42	[***When did the Mother die?***]<br>Date of death<br>Time of death	|___|___| |___|___| |___|___| dd mm yy<br>|___|___| |___|___| hh mm	
1.51	Is the baby alive?	Yes, still alive .... 1<br>No, baby has died 2	→2.01
1.52	When did the baby die?<br>Date of death<br>Time of death	|___|___| |___|___| |___|___| dd mm yy<br>|___|___| |___|___| hh mm	→STOP
**2. Newborn care practices**			
2.01	[***Is this the day 2 visit or the day 6 visit?***]	Day 2 visit .... 1<br>Day 6 visit 2	→2.10
2.02	How long after delivery was the baby given his/her first bath?	|____|____| Hrs<br>[80 if more than 80 hours; 88 if don't know 98 if not done]	→2.10
2.03	Was the water used to bath the baby at room temperature or warm?	Water at room temperature .... 1<br>Warmed water .... 2<br>Don't know 8	
2.10	Is anything being applied to the umbilical stump of the baby?	Yes .... 1<br>No .... 2<br>Don't know 8	 →2.12<br>→2.12

STUDY ID							
1	-					-	

No	Questions	Responses	Skip
2.11	If yes, what is being applied to the cord stump?<br><br>[RECORD ALL REPORTED]	Yes No<br>A. Antibiotic .... 1 .... 2<br>B. Antiseptic .... 1 .... 2<br>C. Turmeric .... 1 .... 2<br>D. Mustard oil .... 1 .... 2<br>E. Chewed rice .... 1 .... 2<br>F. Coconut oil .... 1 .... 2<br>G. Ash .... 1 .... 2<br>H. Cow dung .... 1 .... 2<br>X. Other (Specify)_______________1 .... 2<br>Z. Don't know 1	
2.12	Is the newborn being given massages?	Yes... 1<br>No .... 2<br>Don't know... 8	 →3.01<br>→3.01
2.13	What oil or material is used for the massage?<br><br>[RECORD ALL REPORTED]	Yes No<br>A. Mustard oil .... 1 .... 2<br>B. Sunflower oil .... 1 .... 2<br>C. Coconut oil .... 1 .... 2<br>D. Bukwa .... 1 .... 2<br>X. Other ______________________1 .... 2<br>Z. Don't know 1	
2.14	On average, how many times per day was the baby given the massage in the last 7 days?	|___|___| times per day<br><br>[88: if don't know]	
**3. Feeding Practices**			
3.01	Are you still breastfeeding the baby?	Yes... 1<br>No .... 2<br>Don't know... 8	
3.02	Did the baby have any of the following liquids or foods yesterday during the day or at night?<br><br>Anything else?<br><br>RECORD ALL MENTIONED	Yes No<br>A. Plain water .... 1 .... 2<br>B. Sugar water, honey, or juice .... 1 .... 2<br>C. Infant formula/baby formula .... 1 .... 2<br>D. Cow's or goat's milk .... 1 .... 2<br>E. Tea/infusions .... 1 .... 2<br>F. Other liquid .... 1 .... 2<br>G. Solid/Semi-solid .... 1 .... 2<br>H. Opiates .... 1 .... 2<br>X. Other _____________________ ... 1 .... 2<br><br>Y. Nothing other than BF was given. 1<br>Z. Don't know 1	 <br><br><br><br><br><br><br><br><br><br>→4.01<br>→4.01
3.03	How are you feeding the baby?	Bottle .... 1<br>Spoon .... 2<br>Dropper .... 3<br>Cotton wick .... 4<br>None of the above .... 5<br>Don't know.. 8	

Form 4A: Newborn Routine Follow-up, 1st week

STUDY ID							
1	-					-	

No	Questions	Responses	Skip
**4. Smoke, and Tobacco Use**			
4.01	Do you now smoke cigarette or [local cigarette]?	Yes....1<br>No....2	 →4.03
4.02	How often do you smoke cigarette?	10 or more times every day....1<br>Between 5-9 times every day....2<br>Between 1-4 times every day....3<br>More than once per week....4<br>At least once per week....5<br>Occasionally....6	
4.03	When food is being cooked in the house where is the baby usually stay?	In the same place where food is being cooked....1<br>In the same room where food is being cooked....2<br>Else where....3	
**5. Health Care-seeking History**			
5.01	Did the baby have any complications or illness since the last visit?	Yes....1<br>No....2	 →5.03
5.02	What complications or illness did the baby have?<br><br>[PROBE]<br><br>Anything else	Yes No<br>A. Not feeding well....1....2<br>B. Cough....1....2<br>C. Cold/running nose....1....2<br>D. Rapid/ difficult breathing....1....2<br>E. Convulsions....1....2<br>F. Fever....1....2<br>G. Body was cold....1....2<br>H. Little or no movement....1....2<br>I. Skin pustules....1....2<br>J. Jaundice....1....2<br>K. Umbilicus red or discharging pus. 1....2<br>L. Birth defects....1....2<br>X. Other ____________________ ... 1....2<br><br>Z. Don't know....1	
5.03	[***Review your record (referral book) - was the baby referred to the hospital/health facility in the previous visit?***]	Yes....1<br>No....2	
5.04	Did you seek any health care for the newborn for a complication/illness or in response to the referral made at the last visit?	Yes....1<br>No....2	 →6.01
5.05	Why did you seek health care? For newborn complication/illness or in response to the referral at the last visit or both?	Yes No<br>A. In response to referral by CHW ... 1....2<br>B. Self referred for complication..... 1....2<br>C. Self referred for complication..... 1....2<br>Z.Dont Know....1	

Form 4A: Newborn Routine Follow-up, 1st week

STUDY ID
1 - |__|__|__|__|__| - |__|

No	Questions	Responses	Skip
5.06	From whom did you seek care for the baby?<br><br>Anyone else?<br><br>[PROBE TO IDENTIFY EACH TYPE OF PERSON AND RECORD ALL MENTIONED]	Yes No<br>A. Qualified Doctor .... 1 .... 2<br>B. Nurse .... 1 .... 2<br>C. Midwife...................................... 1 .... 2<br>D. Paramedic [Example: ANM, FWV, MA] 1 .... 2<br>E. Community Health Worker<br>[use local term] .... 1 .... 2<br>F. Traditional birth attendant .... 1 .... 2<br>G. Unqualified (village) Doctor .... 1 .... 2<br>H. Homeopath/AYUSH .... 1 .... 2<br>I. Herbalist/ Spiritual healer............ 1 .... 2<br>X. Other.. 1 .... 2<br>(Specify)_______________________<br>Z. Does not know 1	
5.07	Where did you receive the care from [indicate the medically trained provider in 5.06] for the baby?<br><br>Anywhere else?<br><br>PROBE TO IDENTIFY EACH SOURCE AND RECORD ALL MENTIONED	Yes No<br>A. Hospital [use local description] .... 1 .... 2<br>B. 1st level facility [use local description]1 .... 2<br>C. Outreach/Satellite<br>[use local description]................... 1 .... 2<br>D. Doctor's chamber .... 1 .... 2<br>E. Home.. 1 .... 2<br>F. Other .... 1 .... 2<br>(Specify)_______________________<br>G. Does not know 1	
5.08	[***<u>Check 5.07: is the code A indicated? That is, "hospital"?</u>***]	Yes... 1<br>No 2	 →5.13
5.09	Was this a study hospital?	Yes... 1<br>(Specify)___________________|___|___| No .... 2<br>Don't know... 8	
5.10	Was the baby admitted to the hospital?	Yes... 1<br>No .... 2<br>Don't know... 8	 →5.13<br>→5.13
5.11	On what date was the baby admitted to the hospital?	|___|___| |___|___| |___|___| dd mm yy	
5.12	How many days did the baby stay in the hospital?	|___|___| days<br>[00: if less than 1 day]	
5.13	Has the baby received any medicine at home?	Yes... 1<br>No .... 2<br>Don't know... 8	 →6.01<br>→6.01
5.14	What medicines has the baby received at home?		

Name of Medicine & Code	Still Given	Total days given
____|___|___|	1 Yes 2 No	|___|___|
____|___|___|	1 Yes 2 No	|___|___|

STUDY ID						
1 -						-

No	Questions		Responses		Skip
	____|___|___|		1 Yes 2 No		|___|___|
	____|___|___|		1 Yes 2 No		|___|___|
**6. New born assessment**					
6.01	IS THE BABY PRESENT?		Yes, present .... 1<br>Yes, present but parent refused child assessment .... 2<br>No, baby is absent 3		 <br>→STOP<br>→STOP
	**NOW CONDUCT A COMPLETE CLINICAL ASSESSMENT OF THE BABY AND RECORD FINDINGS BELOW**				
			**SUSPECTED SEPSIS CRITERIA**		
6.02	Respiratory rate<br>If rr ≥60 b/min, count again	|___|___| breaths/min<br>|___|___| breaths/min	**RESPIRATORY RATE ≥60**	**1 Yes**	**2 No**
6.03	Severe chest Indrawing		**SEVERE CHEST IN-DRAWING**	**1 Yes**	**2 No**
6.04	Axillary temperature	|___|___|___|.|___|°F<br>|___|___|.|___|°C	**HIGH AXILLARY TEMPERATURE *≥38.0°C (≥100.4°F)***	**1 Yes**	**2 No**
6.05	*If Temperature (Axillary) is ≥38.0°C (≥100.4°F) or <35.5˚C (<95.9˚F) wait 10 minutes and take again*<br>***Be Careful to Record final Temperature in Correct Space***		**LOW AXILLARY TEMPERATURE *<35.5˚C (<95.9˚F)***	**1 Yes**	**2 No**
6.06	Level of consciousness of the baby and movement	Normal movement .... 1<br>Movement only on stimulation ...... 2<br>No movement at all or unconscious 3	**NO MOVEMENT or MOVEMENT ONLY ON STIMULATION** **(circle "Yes" if reported 2 or 3 on left)**	**1 Yes**	**2 No**
6.07	Convulsions	Reported convulsions .... 1<br>Observed convulsions .... 2<br>No convulsion 3	**CONVULSION** (circle "Yes" if reported 2 or 3 on left)	**1 Yes**	**2 No**
6.08	***Ask mother whether the baby is feeding well or not***<br>Reports<br>Difficulty in Feeding .... 1<br>No difficulty in feeding .... 2<br>***If feeding difficulty reported, perform feeding assessment by observing breast feeding***<br>Poor Well<br>Position .... 1 .... 2<br>Attachment .... 1 .... 2<br>***Help mother to improve position & attachment and observe breast feeding***<br>Poor Well<br>Attachment .... 1 .... 2<br>Sucking .... 1 .... 2<br>***Report as a poor feeding if baby still has poor attachment or poor sucking***		**POOR FEEDING**	**1 Yes**	**2 No**

STUDY ID							
**1** -						-	

No	Questions	Responses	Skip
6.11	Skin pustules	**1 Yes 2 No**	
6.12	Umbilicus red or discharging pus	1 None 2 Redness present 3 Discharging pus 4 Both present	
6.13	Is the baby suffering from jaundice?	1 No Jaundice 2 Jaundice 3 Severe Jaundice	
6.14	Any other complications?	**1 Yes 2 No**	
	Specify ____		
6.15	Eligible for screening for suspected sepsis<br>*[Child will be eligible for screening by physician for suspected sepsis if any of the criteria for suspected sepsis are met (any of the shaded area in 6.02-6.08. is "Yes")]*	**1 Yes 2 No**	
**7. Visit outcome**			
7.01	[***Was the baby referred? if yes, where?***]	Yes, to "study" facility .... 1<br>(specify)____________________|___|___| Yes, to mobile study clinical team .... 2<br>(specify)____________________|___|___| No 3	→STOP
7.02	[***What is the reason for referral?***]	Suspected sepsis .... 1<br>Other illness .... 2<br>(specify)____________________|___|___|	
7.03	[***Did the caregiver accept referral?***]	Yes... 1<br>No 2	→STOP
If caregiver did not accept the referral, visit next day, reinforce referral and complete form-5 for that visit			
7.04	[***Why did the caregiver refuse referral?***] |___|___| ____<br>____		
