# Supplementary material for: Association of clinical signs of possible serious bacterial infections identified by community health workers with mortality of young infants in South Asia: a prospective, observational cohort study
Source: eClinicalMedicine. 2025 Jan 18;80:103070. doi: 10.1016/j.eclinm.2025.103070 (PMC11787667; doi:10.1016/j.eclinm.2025.103070)
Supplement: ANISA_Data-Form-4B_FuV-7-59days_version 6.0_06.06 [file mmc2.pdf]

Form 4B: Newborn Routine Follow-up 8-59 days

| STUDY ID | | | | | | |
|---|---|---|---|---|---|---|
| 1 | - | | | | | - | |

| 4B | CLUSTER CODE \|___\|___\|___\| |
|---|---|

| Scheduled Visit | | | |
|---|---|---|---|
| Day 13☐ | Day 20☐ | Day 27☐ | ANISA |
| Day 34☐ | Day 41☐ | Day 48☐ | Day 59☐ |

**This form is to be completed for all newborns enrolled in surveillance, for the scheduled visits after the first week after birth, i,e., day 13 through day 59. In case the newborn is not present on the scheduled visit date, one re-visit may be attempted**

| **1. Address and identification information** | | | |
|---|---|---|---|
| 1.01 | **Country/Site** | **BANGLADESH/ SYLHET** | **1** |
| 1.02 | **Upazila** | | |
| 1.03 | **Union** | | |
| 1.04 | **Village** | | |
| 1.05 | **Bari** | | |
| 1.06 | **Household** | | |
| 1.11 | Woman's Current ID | _ _ _ _ - _ _ _ - _ _ - _ _ | |
| 1.12 | Woman's Permanent ID | _ _ _ _ - _ _ _ - _ _ - _ _ | |
| 1.13 | Woman's Name | | |
| 1.14 | Husband's Name | | |
| 1.15 | Household Head's Name | | |
| 1.16 | CHW's Name & Code | | |
| 1.21 | Date of Visit (1st attempt) | | |
| 1.22 | Date of Visit (2nd attempt) | | |
| 1.24 | Time of interview | Start time \|__\|__\|: \|__\|__\| h h m m End time \|__\|__\|: \|__\|__\| h h m m | |

| STUDY ID | | | | | | | |
|---|---|---|---|---|---|---|---|
| 1 | - | | | | | - | |

| No | Questions | Responses | Skip |
|---|---|---|---|
| *1.31* | [***Visit Outcome***] | Completed ........................................ 1<br>Incomplete........................................ 2<br>Refused follow-up.................................. 3<br>No-one present to respond after 2 visits..... 4<br>Out-migrated ........................................ 5 | <br><br>→STOP<br>→STOP<br>→STOP |
| *1.32* | [***Respondent***] | Mother ........................................ 1<br>Grand Mother........................................ 2<br>Aunt ........................................ 3<br>Sister ........................................ 4<br>Father ........................................ 5<br>Grand father ........................................ 6<br>Uncle........................................ 8<br>Brother ........................................ 9<br>Other (Specify__________________ .......... 7 | |
| *1.41* | [***Is the Mother alive?***] | Yes, Mother is alive.................................. 1<br>No, Mother has died.................................. 2 | →1.51 |
| *1.42* | [***When did the Mother die?***]<br>Date of death<br>Time of death | \|___\|___\| \|___\|___\| \|___\|___\|<br>dd mm yy<br>\|___\|___\| \|___\|___\|<br>hh mm | |
| *1.51* | Is the baby alive? | Yes, still alive........................................ 1<br>No, baby has died .................................. 2 | →2.01 |
| *1.52* | When did the baby die?<br>Date of death<br>Time of death | \|___\|___\| \|___\|___\| \|___\|___\|<br>dd mm yy<br>\|___\|___\| \|___\|___\|<br>hh mm | →STOP |
| **2. Newborn care practices** | | | |
| *2.01* | Is the newborn being given a massage? | Yes ........................................ 1<br>No ........................................ 2<br>Don't know ........................................ 8 | <br>→3.01<br>→3.01 |
| *2.02* | What oil or material used for the massage?<br><br>[RECORD ALL REPORTED] | Yes No<br>A. Mustard oil ............................ 1........ 2<br>B. Sunflower oil............................ 1........ 2<br>C. Coconut oil............................ 1........ 2<br>D. Bukwa............................ 1........ 2<br>X. Other ______________________1........ 2<br>Z. Don't know............................ 1 | |
| *2.03* | On average, how many times per day was the baby given the massage in the last 7 days? | \|___\|___\| times per day<br>[88: if don't know] | |

| STUDY ID | | | | | | | |
|---|---|---|---|---|---|---|---|
| **1** - | | | | | | - | |

| No | Questions | Responses | Skip |
|---|---|---|---|
| **3. Feeding Practices** | | | |
| *3.01* | Are you still breastfeeding the baby? | Yes .......... 1<br>No .......... 2<br>Don't know .......... 8 | |
| *3.02* | Did the baby have any of the following liquids or foods yesterday during the day or at night?<br><br>Anything else?<br><br>RECORD ALL MENTIONED | Yes No<br>A. Plain water .......... 1........2<br>B. Sugar water, honey, or juice........ 1........2<br>C. Infant formula/baby formula ...... 1........2<br>D. Cow's or goat's milk .......... 1........2<br>E. Tea/infusions .......... 1........2<br>F. Other liquid .......... 1........2<br>G. Solid/Semi-solid .......... 1........2<br>X. Other ____________________ ... 1........2<br><br>Y. Nothing other than BF was given 1<br>Z. Don't know.......... 1 | |
| *3.03* | How are you feeding the baby? | Bottle .......... 1<br>Spoon .......... 2<br>Dropper .......... 3<br>Cotton wick .......... 4<br>None of the above .......... 5<br>Don't know .......... 8 | |
| **4. Smoke, and Tobacco Use** | | | |
| *4.01* | [***Is this the day 27 visit?***] | Day 27 visit.......... 1<br>Other visit .......... 2 | <br>→5.01 |
| *4.02* | Do you now smoke cigarette or [local cigarette]? | Yes .......... 1<br>No .......... 2 | <br>→4.04 |
| *4.03* | How often do you smoke cigarette? | 10 or more times every day.......... 1<br>Between 5-9 times every day .......... 2<br>Between 1-4 times every day .......... 3<br>More than once per week .......... 4<br>At least once per week .......... 5<br>Occasionally .......... 6 | |
| *4.04* | When food is being cooked in the house where is the baby usually stay? | In the same place where food is being cooked .......... 1<br>In the same room where food is being cooked .......... 2<br>Else where .......... 3 | |
| **5. Health Care-seeking History** | | | |
| *5.01* | Did the baby have any complications or illness since the last visit? | Yes.......... 1<br>No .......... 2 | <br>→5.03 |

| STUDY ID | | | | | | | |
|---|---|---|---|---|---|---|---|
| 1 | - | | | | | - | |

| No | Questions | Responses | Skip |
|---|---|---|---|
| *5.02* | What complications or illness did the baby have?<br><br>[PROBE]<br><br>Anything else | Yes No<br>A. Not feeding well ........................ 1........2<br>B. Cough........................................ 1........2<br>C. Cold/running nose ...................... 1........2<br>D. Rapid/ difficult breathing ............ 1........2<br>E. Convulsions................................ 1........2<br>F. Fever .......................................... 1........2<br>G. Body was cold ............................ 1........2<br>H. Little or no movement ............... 1........2<br>I. Skin pustules............................... 1........2<br>J. Jaundice...................................... 1........2<br>K. Umbilicus red or discharging pus. 1........2<br>L. Birth defects............................... 1........2<br>X. Other ____________________ ... 1........2<br><br>Z. Don't know.................................. 1 | |
| *5.03* | [***Review your record (referral book) - was the baby referred to the hospital/health facility in the previous visit?***] | Yes............................................................ 1<br>No ............................................................ 2 | |
| *5.04* | Did you seek any health care for the newborn for a complication/illness or in response to the referral made at the last visit? | Yes............................................................ 1<br>No ............................................................ 2 | <br>→6.01 |
| *5.05* | Why did you seek health care? For newborn complication/illness or in response to the referral at the last visit or both? | Yes No<br>A. In response to referral by CHW ... 1........2<br>B. Self referred for complication..... 1........2<br>C. Self referred for complication..... 1........2<br>Z.Dont Know ................................... 1 | |
| *5.06* | From whom did you seek care for the baby?<br><br>Anyone else?<br><br>[PROBE TO IDENTIFY EACH TYPE OF PERSON AND RECORD ALL MENTIONED] | Yes No<br>A. Qualified Doctor ......................... 1........2<br>B. Nurse ........................................ 1........2<br>C. Midwife...................................... 1........2<br>D. Paramedic [Example: ANM, FWV, MA] 1........2<br>E. Community Health Worker<br>[use local term] ....................... 1........2<br>F. Traditional birth attendant (TBA). 1........2<br>G. Unqualified (village) Doctor ........ 1........2<br>H. Homeopath/AYUSH.................... 1........2<br>I. Herbalist/ Spiritual healer............ 1........2<br>X. Other.......................................... 1........2<br>(Specify)______________________<br>Z. Does not know ............................ 1 | |
| *5.07* | Where did you receive the care from [indicate the medically trained provider in 5.06] for the baby?<br><br>Anywhere else?<br><br>PROBE TO IDENTIFY EACH SOURCE AND RECORD ALL MENTIONED | Yes No<br>A. Hospital [use local description] .......... 1........2<br>B. 1st level facility [use local description]1........2<br>C. Outreach/Satellite<br>[use local description]................... 1........2<br>D. Doctor's chamber ....................... 1........2<br>E. Home ......................................... 1........2<br>F. Other.......................................... 1........2<br>(Specify)______________________<br>G. Does not know ........................... 1 | |

| STUDY ID | | | | | | | |
|---|---|---|---|---|---|---|---|
| 1 | - | | | | | - | |

| No | Questions | Responses | Skip |
|---|---|---|---|
| *5.08* | [***Check 5.07: is the code A indicated? That is, "hospital"?***] | Yes.......... 1<br>No .......... 2 | <br>→5.13 |
| *5.09* | Was this a study hospital? | Yes.......... 1<br>(Specify)____________ \|___\|___\|<br>No .......... 2<br>Don't know .......... 8 | |
| *5.10* | Was the baby admitted to the hospital? | Yes.......... 1<br>No .......... 2<br>Don't know .......... 8 | <br>→5.13<br>→5.13 |
| *5.11* | On what date was the baby admitted to the hospital? | \|___\|___\| \|___\|___\| \|___\|___\|<br>dd mm yy | |
| *5.12* | How many days did the baby stay in the hospital? | \|___\|___\| days<br>[00: if less than 1 day] | |
| *5.13* | Has the baby received any medicine at home? | Yes.......... 1<br>No .......... 2<br>Don't know .......... 8 | <br>→6.01<br>→6.01 |
| *5.14* | What medicines has the baby received at home? | | |

| Name of Medicine & Code | Still Given | Total days given |
|---|---|---|
| ____________________ \|___\|___\| | 1 Yes 2 No | \|___\|___\| |
| ____________________ \|___\|___\| | 1 Yes 2 No | \|___\|___\| |
| ____________________ \|___\|___\| | 1 Yes 2 No | \|___\|___\| |
| ____________________ \|___\|___\| | 1 Yes 2 No | \|___\|___\| |

| No | Questions | Responses | Skip |
|---|---|---|---|
| **6. Maternal Nutritional Status** | | | |
| *6.01* | [***Is this the day 59 visit?***] | Day 59 visit .......... 1<br>Other visit .......... 2 | <br>→8.01 |
| *6.02* | Mid-upper Arm circumference | \|___\|___\|.\|___\| cms | |
| *6.03* | Weight | \|___\|___\|___\| . \|___\| kgs | |
| **7. Newborn Immunization History** | | | |
| *7.01* | Has the baby received any vaccination? | Yes .......... 1<br>No .......... 2 | <br>→8.01 |
| *7.02* | Can you show me the baby's immunization card? | Yes .......... 1<br>No .......... 2 | <br>→8.01 |
| *7.03* | [***Record dates of immunization from the immunization card***] | | |
| | BCG | \|___\|___\| \|___\|___\| \|___\|___\| | |
| | OPV 0 | \|___\|___\| \|___\|___\| \|___\|___\| | |

| STUDY ID | | | | | | | |
|---|---|---|---|---|---|---|---|
| 1 | - | | | | | - | |

| No | Questions | Responses | | | Skip |
|---|---|---|---|---|---|
| | OPV 1 | \|___\|___\| | \|___\|___\| | \|___\|___\| | |
| | DPT 1 or HepB+DPT 1 or Pentavalent | \|___\|___\| | \|___\|___\| | \|___\|___\| | |
| | PCV (Momin qazi will provide this) | \|___\|___\| dd | \|___\|___\| mm | \|___\|___\| yy | |

**8. New born assessment**

| No | Questions | Responses | Skip |
|---|---|---|---|
| *8.01* | [***Is the baby present?***] | Yes, present ............ 1<br>Yes, present but parent refused child assessment ............ 2<br>No, baby is absent ............ 3 | <br><br>→STOP<br>→STOP |

**NOW CONDUCT A COMPLETE CLINICAL ASSESSMENT OF THE BABY AND RECORD FINDINGS BELOW**

| No | Questions | Responses | SUSPECTED SEPSIS CRITERIA | |
|---|---|---|---|---|
| *8.02* | Respiratory rate<br>If rr ≥60 b/min, count again | \|___\|___\| breaths/min<br>\|___\|___\| breaths/min | **RESPIRATORY RATE ≥60** | **1 Yes 2 No** |
| *8.03* | Severe chest Indrawing | | **SEVERE CHEST IN-DRAWING** | **1 Yes 2 No** |
| *8.04* | Axillary temperature | \|___\|___\|___\|.\|___\|°F<br>\|___\|___\|.\|___\|°C | **HIGH AXILLARY TEMPERATURE *≥38.0°C (≥100.4°F)*** | **1 Yes 2 No** |
| *8.05* | *If Temperature (Axillary) is ≥38.0°C (≥100.4°F) or <35.5˚C (<95.9˚F) wait 10 minutes and take again*<br>***Be Careful to Record final Temperature in Correct Space*** | | **LOW AXILLARY TEMPERATURE *<35.5˚C (<95.9˚F)*** | **1 Yes 2 No** |
| *8.06* | Level of consciousness of the baby and movement | Normal movement ............ 1<br>Movement only on stimulation ...... 2<br>No movement at all or unconscious ............ 3 | **NO MOVEMENT or MOVEMENT ONLY ON STIMULATION** (circle "Yes" if reported 2 or 3 on left ) | **1 Yes 2 No** |
| *8.07* | Convulsions | Reported convulsions ............ 1<br>Observed convulsions ............ 2<br>No convulsions ............ 3 | **CONVULSION** | **1 Yes 2 No** |
| *8.08* | ***Ask mother whether the baby is feeding well or not***<br>Reports<br>Difficulty in Feeding ............ 1<br>No difficulty in feeding ............ 2<br>***If feeding difficulty reported, perform feeding assessment by observing breast feeding***<br>Poor Well<br>Position ............ 12 Attachment 1 ............ 2<br>***Help mother to improve position & attachment and observe breast feeding***<br>Poor Well<br>Attachment ............ 1...........2<br>Sucking ............ 1...........2<br>***Report as a poor feeding if baby still has poor attachment or poor sucking*** | | **POOR FEEDING** | **1 Yes 2 No** |

| STUDY ID | | | | | | | |
|---|---|---|---|---|---|---|---|
| **1** | - | | | | | - | |

| No | Questions | Responses | Skip |
|---|---|---|---|
| *8.11* | Skin pustules | **1 Yes 2 No** | |
| *8.12* | Umbilicus red or discharging pus | 1 None 2 Redness present 3 Discharging pus 4 Both present | |
| *8.13* | Is the baby suffering from jaundice? | 1 No Jaundice 2 Jaundice 3 Severe Jaundice | |
| *8.14* | Any other complications? | **1 Yes 2 No** | |
| | Specify ________________________________________ | | |
| 8.15 | Eligible for screening for suspected sepsis<br>*[Child will be eligible for screening by physician for suspected sepsis if any of the criteria for suspected sepsis are met (If any of the shaded area in 8.02-8.08. is "Yes")]* | **1 Yes 2 No** | |
| **9. Visit outcome** | | | |
| *9.01* | [***Was the baby referred? if yes, where?***] | Yes, to "study" facility ................................1<br>(specify)____________________\|___\|___\|<br>Yes, to mobile study clinical team................2<br>(specify)____________________\|___\|___\|<br>No...............................................................3 | →STOP |
| *9.02* | [***What is the reason for referral?***] | Suspected sepsis ........................................1<br>Other illness ................................................2<br>(specify)____________________\|___\|___\| | |
| *9.03* | [***Did the caregiver accept referral?***] | Yes...............................................................1<br>No................................................................2 | →STOP |
| If caregiver did not accept the referral, visit next day, reinforce referral and complete form-5 for that visit | | | |
| *9.04* | [***Why did the caregiver refuse referral?***]<br>________________________________________<br>________________________________________ | | \|___\|___\| |
