# Supplementary material for: Association of clinical signs of possible serious bacterial infections identified by community health workers with mortality of young infants in South Asia: a prospective, observational cohort study
Source: eClinicalMedicine. 2025 Jan 18;80:103070. doi: 10.1016/j.eclinm.2025.103070 (PMC11787667; doi:10.1016/j.eclinm.2025.103070)
Supplement: ANISA_Data-Form-6_P_version 6.0_06.06 [file mmc3.pdf]

Form 6: Physician's Assessment

STUDY ID							
1	-					-	

6	CLUSTER CODE |___|___|___|

Suspected Infection Case ☐
Healthy Control Case ☐

ANISA

This form is to be completed for all newborns with suspected sepsis/healthy control seen by a study physician.

**1. Address and identification information**			
1.01	**Country/Site**	**BANGLADESH/ SYLHET**	**1**
1.02	**Upazila**		
1.03	**Union**		
1.04	**Village**		
1.05	**Bari**		
1.06	**Household**		
1.11	Woman's Current ID	_ _ _ _ - _ _ _ - _ _ - _ _	
1.12	Woman's Permanent ID	_ _ _ _ - _ _ _ - _ _ - _ _	
1.13	Woman's Name		
1.14	Husband's Name		
1.15	Household Head's Name		
1.16	CHW's Name & Code		
1.21	Date of Visit		
1.24	Time of interview	Start time |__|__|: |__|__| h h m m End time |__|__|: |__|__| h h m m	
1.18	Physician's Name		
1.19	Location	☐ **Home**	
		☐ **Facility:**	
1.20	Selected as Sepsis Case or Health Control	☐ **Sepsis case**	
		☐ **Healthy control**	
		☐ **Neither**	

STUDY ID
1 - _ _ _ _ _ - _

1.31	[***Caretaker (relationship to newborn)***]	Mother .... 1<br>Grand Mother .... 2<br>Aunt .... 3<br>Sister .... 4<br>Father... 5<br>Grand father .... 6<br>Uncle .... 8<br>Brother... 9<br>Other (Specify___________________.......... 7
1.32	Was the newborn referred by the CHW or did someone else referred or was it a self-referral?	Referred by CHW .... 1<br>Referred by someone else .... 2<br>Self referred 3

**2. History of infant's symptoms (Ask the Caretaker)**				
2.01	Has the baby been feeding poorly?	1 Yes	2 No	8 Don't know
2.02	Has the baby had a fever?	1 Yes	2 No	8 Don't know
2.03	Has the baby felt cold to the touch?	1 Yes	2 No	8 Don't know
2.04	Has the baby had a runny nose recently?	1 Yes	2 No	8 Don't know
2.05	Does the baby have a cough?	1 Yes	2 No	8 Don't know
2.06	Does the baby have fast or difficult breathing?	1 Yes	2 No	8 Don't know
2.07	Has the baby had vomiting?	1 Yes	2 No	8 Don't know
2.08	Has the baby been having diarrhea?	1 Yes	2 No	8 Don't know
2.09	Has there been blood in the baby's stools?	1 Yes	2 No	8 Don't know
2.10	Has there been discharge from the baby's umbilicus?	1 Yes	2 No	8 Don't know
2.11	Other, specify____|___|___|			
2.12	Other, specify____|___|___|			
2.13	Other, specify____|___|___|			
**3. Medication History (Ask the Caretaker)**				
3.01	Did the baby receive any medicine over the last seven days?	1 Yes	2 No	8 Don't know

3.20	What medicines were received by the baby?				
	Name of Medicine & Code	Types	Times /day	Still Given	Total days given
	______________________________|___|___|	1 Inj 2 Liq 3 Tab	|___|	1 Yes 2 No	|___|___|
	______________________________|___|___|	1 Inj 2 Liq 3 Tab	|___|	1 Yes 2 No	|___|___|
	______________________________|___|___|	1 Inj 2 Liq 3 Tab	|___|	1 Yes 2 No	|___|___|
	______________________________|___|___|	1 Inj 2 Liq 3 Tab	|___|	1 Yes 2 No	|___|___|

STUDY ID							
1	-					-	

4. Infant Clinical assessment			
Assessment			MEET INCLUSION CRITERIA
4.01	Respiratory rate |___|___| breaths/min<br>If rr ≥60 b/min, count again |___|___| breaths/min		**RESPIRATORY RATE ≥60** 1 Yes 2 No
4.02	Severe chest Indrawing		**SEVERE CHEST IN-DRAWING** 1 Yes 2 No
4.03	Axillary temperature	|___|___|___|.|___|°F<br>|___|___|.|___|°C	**HYPERTHERMIA** ***≥38.0°C (≥100.4°F)*** 1 Yes 2 No
4.04	*If Temperature (Axillary) is ≥38.0°C (≥100.4°F) or <35.5˚C (<95.9˚F) wait 10 minutes and take again*<br>***Be Careful to Record final Temperature in Correct Space***		**HYPOTHERMIA** *<35.5˚C (<95.9˚F)* 1 Yes 2 No
4.05	Level of consciousness of the baby and movement	Normal movement.........................1<br>Movement only on stimulation ......2<br>No movement at all/unconscious...3	**NO MOVEMENT or MOVEMENT ONLY ON STIMULATION** **(circle "Yes" if reported 2 or 3 on left)** 1 Yes 2 No
4.06	Convulsions	Reported convulsions ....1<br>Observed convulsions....................2<br>No convulsion 3	**CONVULSIONS** (circle "Yes" if reported 2 or 3 on left) 1 Yes 2 No
4.07	Feeding assessment performed by the	Physician .... 1<br>Nurse... 2<br>CHW 3	**POOR FEEDING** 1 Yes 2 No
4.11	Skin pustules		None ....1<br>Some skin pustules ....2<br>Many or severe skin pustules 3
4.12	Is there pus (white or yellow) discharge present in the umbilicus?		No discharge present...1<br>Thin clear discharge present...................................2<br>White or yellow discharge present..........................3
4.13	Is the cord or the base of umbilical stump red?		No redness...1<br>Redness limited to stump of cord...........................2<br>Redness extending to skin surrounding stump but less than 1 finger wide.......................................3<br>Redness extending to skin surrounding stump one finger or more wide...4
4.14	Is the baby suffering from jaundice		No jaundice present ....1<br>Mild jaundice ....2<br>Moderate jaundice ....3<br>Severe Jaundice 4
	Presence of congenital anomalies		

STUDY ID
1 - _ _ _ _ _ - _

4.15	if yes, specify ____|___|___|	
4.16	Apnoea	**1 Yes 2 No**
4.17	Cyanosis	**1 Yes 2 No**
4.18	Unable to cry	**1 Yes 2 No**
4.19	Bulging fontanelle	**1 Yes 2 No**
4.20	Prolonged capillary refill	**1 Yes 2 No**
4.21	Persistent vomiting	**1 Yes 2 No**
4.22	Diarrhoea	**1 Yes 2 No**
4.23	Runny nose	**1 Yes 2 No**
4.24	Conjunctivitis	**1 Yes 2 No**
4.25	Wheeze	**1 Yes 2 No**
4.26	White patches in mouth	**1 Yes 2 No**
4.27	Diaper dermatitis	**1 Yes 2 No**
4.28	Any other sign? Specify ____|____|____|	
4.29	Any other sign? Specify ____|____|____|	
**5.00 Exclusion Criteria**		
5.01	Hospitalized for at least one night in the preceding 7 days (except post-natal hospital stays of facility-born infants) [FROM HOSPITAL RECORDS OR ANISA DATA BASE]	**1 Yes 2 No**
5.02	Previously enrolled with an episode of suspected serious infection in the ANISA study in the preceding 7 days [FROM HOSPITAL RECORDS OR ANISA DATA BASE]	**1 Yes 2 No**
**5.10 Eligibility for enrollment**		
5.11	Meet **Inclusion criteria** (any of 4.01 to 4.07 is "Yes")	**1 Yes 2 No**
5.12	**Exclusion criteria** present (any of 5.01 OR 5.02 is "Yes")	**1 Yes 2 No**
5.13	Whether the child previously selected as **Healthy Control**	**1 Yes 2 No**

Child will be eligible for the enrollment

a. As Sepsis case if any Inclusion criteria is met (5.11 is "Yes") and there are no Exclusion criteria (5.12 is "No")

As Healthy Control if Child had been previously selected as Healthy control (5.13 is "Yes") and the child does NOT meet any Inclusion (5.11 is "No") and Exclusion criteria (5.12 is "No")

**6.00 Enrollment for the sepsis surveillance**			
6.01	Consent given for specimen collection	1 Yes	2 No
6.02	Child enrolled for specimen collection	1 Yes	2 No

STUDY ID							
1	-					-	

6.11	Blood specimen collected?	Yes .... 1 →6.21<br>No 2
6.12	Reason of not specimen collected	1 Attempt failed 2 Consent withdrawn
6.21	NP/OP specimen collected?	Yes .... 1 →6.31<br>No 2
6.22	Reason of not specimen collected	1 Attempt failed 2 Consent withdrawn
6.31	CSF specimen collected?	Yes .... 1 →7.01<br>No 2
6.32	Reason of not specimen collected	1 Attempt failed 2 Consent withdrawn

7. Patient Management

7.01	Physician's diagnosis ________________ |____|____|		
7.02	Physician's recommendation?	To admit .... 1<br>To refer .... 2 →7.05<br>For home treatment .... 3 →7.11	
7.03	Did the caregiver accept admission?	Yes .... 1 →7.11<br>No 2	
7.04	Why did the caregiver refuse admission? |___|___|→7.11<br>________________		
7.05	Did the caregiver accept referral?	Yes .... 1 →7.11<br>No 2	
7.06	Why did the caregiver refuse referral? |___|___| ________________		
7.11	Medicine given by **STUDY physician** (If yes, then provide details below) 1 Yes 2 No		
	**Drug Name and Code**	**Start(dd / mm / yy)**	**End / mm / yy)**
7.12	____________<br>____________|___|___|	|___|___|/|___|___|/|___|___|	|___|___|/|___|___|/|___|___|
7.13	____________<br>____________|___|___|	|___|___|/|___|___|/|___|___|	|___|___|/|___|___|/|___|___|
7.14	____________<br>____________|___|___|	|___|___|/|___|___|/|___|___|	|___|___|/|___|___|/|___|___|
