# Supplementary material for: Association of clinical signs of possible serious bacterial infections identified by community health workers with mortality of young infants in South Asia: a prospective, observational cohort study
Source: eClinicalMedicine. 2025 Jan 18;80:103070. doi: 10.1016/j.eclinm.2025.103070 (PMC11787667; doi:10.1016/j.eclinm.2025.103070)
Supplement: Supplementary Figure and Tables [file mmc4.docx]

**Association of clinical signs of possible serious bacterial infections identified by community health workers with mortality of young infants in South Asia: a prospective, observational cohort study**

Gary L. Darmstadt, Saifuddin Ahmed, Mohammad Shahidul Islam, Safa Abdalla, Shams El Arifeen, Melissa L Arvay, Abdullah H Baqui, Zulfiqar A Bhutta, Anuradha Bose, Nicholas E Connor, Belal Hossain, Rita Isaac, Arif Mahmud, Dipak K Mitra, Luke C Mullany, Imran Nisar, Kalpana Panigrahi, Pinaki Panigrahi^,^ Qazi Sadeq-ur Rahman, Senjuti Saha, Sajid B Soofi, Nardos Solomon, Mathuram Santosham, Stephanie J Schrag, Shamim A Qazi, Samir K Saha

**Supplemental Data**

**Table S1. Pairwise overlap between signs of possible serious bacterial infection identified by community health workers in infants ages 0-<3 days**

| Sign (total)^a^ | Fast breathing | Chest indrawing | Fever | Hypothermia | No movement | Convulsions | Poor feeding |
| --- | --- | --- | --- | --- | --- | --- | --- |
| Fast breathing (N=3,089) | 3,089 (100.0%) | 303  (9.8%) | 366 (11.8%) | 167  (5.4%) | 171  (5.5%) | 136  (4.4%) | 456  (14.8%) |
| Chest indrawing (N=410) | 3  (73.9%) | 410  (100.0%) | 39 (9.5%) | 58  (14.1%) | 71  (17.3%) | 21  (5.1%) | 126  (31.0%) |
| Fever (N=1,029) | 366  (35.6%) | 39  (3.8%) | 1,029 (100.0%) | 0  (0.0%) | 34  (3.3%) | 37  (3.6%) | 76  (7.4%) |
| Hypothermia (N=1,045) | 167  (16.0%) | 58  (5.6%) | 0  (0.0%) | 1,045 (100.0%) | 192  (18.4%) | 29  (2.8%) | 367  (35.1%) |
| No movement (N=491) | 171  (34.8%) | 71  (14.5%) | 34 (6.9%) | 192  (39.1%) | 491  (100.0%) | 77  (15.7%) | 354  (72.2%) |
| Convulsions (N=325) | 136  (41.8%) | 21  (6.5%) | 37 (11.4%) | 29  (8.9%) | 77  (23.7%) | 325 (100.0%) | 139  (43.0%) |
| Poor feeding (N=1,532) | 456  (29.8%) | 126  (8.2%) | 76 (5.0%) | 367  (24.0%) | 354  (23.1%) | 139  (9.1%) | 1,532 (100.0%) |

^a^Fast breathing (=respiratory rate >60 breaths per minute), chest indrawing (=severe chest indrawing), fever (=temperature > 38^o^C), hypothermia (=temperature <35.5 ^o^C),

no movement (=no movement or movement only on stimulation), convulsions (by maternal report), poor feeding (=not able to feed at all or stopped feeding well)

**Table S2. Pairwise overlap between signs of possible serious bacterial infection identified by community health workers**

**in infants ages 0-<7 days**

| Sign (total)^a^ | Fast breathing | Chest indrawing | Fever | Hypothermia | No movement | Convulsions | Poor feeding |
| --- | --- | --- | --- | --- | --- | --- | --- |
| Fast breathing (N=4,295) | 4,295 (100.0%) | 407  (9.5%) | 480 (11.2%) | 183  (4.3%) | 212  (4.9%) | 168  (3.9%) | 546  (12.8%) |
| Chest indrawing (N=555) | 407  (73.3%) | 555  (100.0%) | 57 (10.3%) | 66  (11.9%) | 84  (15.1%) | 30  (5.4%) | 152  (27.5%) |
| Fever (N=1,440) | 480  (33.3%) | 57  (4.0%) | 1,440 (100.0%) | 0  (0.0%) | 52  (3.6%) | 50  (3.5%) | 114  (7.9%) |
| Hypothermia (N=1,266) | 183  (14.5%) | 66  (5.2%) | 0  (0.0%) | 1,266 (100.0%) | 242  (19.1%) | 31  (2.4%) | 432  (34.1%) |
| No movement (N=652) | 212  (32.5%) | 84  (12.9%) | 52 (8.0%) | 242  (37.1%) | 652  (100.0%) | 102  (15.6%) | 456  (70.0%) |
| Convulsions (N=454) | 168  (37.0%) | 30  (6.6%) | 50 (11.0%) | 31  (6.8%) | 102  (22.5%) | 454 (100.0%) | 176  (39.0%) |
| Poor feeding (N=1,928) | 546  (28.3%) | 152  (7.9%) | 114 (5.9%) | 432  (22.4%) | 456  (23.7%) | 176  (9.1%) | 1,928 (100.0%) |

^a^Fast breathing (=respiratory rate >60 breaths per minute), chest indrawing (=severe chest indrawing), fever (=temperature > 38^o^C), hypothermia (=temperature <35.5 ^o^C),

no movement (=no movement or movement only on stimulation), convulsions (by maternal report), poor feeding (=not able to feed at all or stopped feeding well)

**Table S3. Pairwise overlap between signs of possible serious bacterial infection identified by community health workers**

**in infants ages 7-<60 days**

| Sign (total)^a^ | Fast breathing | Chest indrawing | Fever | Hypothermia | No movement | Convulsions | Poor feeding |
| --- | --- | --- | --- | --- | --- | --- | --- |
| Fast breathing (N=4,091) | 4,091 (100.0%) | 917  (22.4%) | 288 (7.0%) | 27  (0.6%) | 102  (2.5%) | 59  (1.4%) | 300  (7.3%) |
| Chest indrawing (N=1,636) | 917  (56.0%) | 1,636  (100.0%) | 89  (5.5%) | 21  (1.2%) | 36  (2.0%) | 26  (1.5%) | 100  (5.8%) |
| Fever (N=948) | 288  (30.4%) | 89  (9.4%) | 948 (100.0%) | 0  (0.0%) | 85  (9.0%) | 23  (2.4%) | 211  (22.2%) |
| Hypothermia (N=321) | 27  (8.4%) | 21  (6.5%) | 0  (0.0%) | 321  (100.0%) | 68  (21.2%) | 15  (4.7%) | 90  (28.9%) |
| No movement (N=389) | 102  (26.2%) | 36  (9.3%) | 85 (21.9%) | 68  (17.4%) | 389  (100.0%) | 24  (6.2%) | 288  (74.0%) |
| Convulsions (N=381) | 59  (15.5%) | 26  (6.8%) | 23  (6.0%) | 15  (3.9%) | 24  (6.3%) | 381 (100.0%) | 37  (9.7%) |
| Poor feeding (N=1,155) | 300  (26.0%) | 100  (8.7%) | 211 (18.3%) | 90  (7.8%) | 288  (24.9%) | 37  3.2%) | 1,155  (100.0%) |

^a^Fast breathing (=respiratory rate >60 breaths per minute), chest indrawing (=severe chest indrawing), fever (=temperature > 38^o^C), hypothermia (=temperature <35.5 ^o^C),

no movement (=no movement or movement only on stimulation), convulsions (by maternal report), poor feeding (=not able to feed at all or stopped feeding well)

**Table S4: Kaplan-Meier mortality probabilities by signs of possible serious bacterial**

**infection (PSBI)^a,b^ identified by community health workers among 63,017 young infants**

**ages 0-<60 days**

|  |  | **Mortality probabilities** | | | |
| --- | --- | --- | --- | --- | --- |
| **^a^Sign** | **Age (days)** | **No sign** | **Only this sign** | **With other sign(s)** | **Only other sign(s)** |
| Fast breathing | 1 | 0.002 | 0.018 | 0.125 | 0.162 |
|  | 2 | 0.004 | 0.029 | 0.262 | 0.217 |
|  | 7 | 0.008 | 0.058 | 0.431 | 0.341 |
|  | 28 | 0.014 | 0.078 | 0.481 | 0.463 |
|  | 59 | 0.020 | 0.125 | 0.560 | 0.511 |
| Chest indrawing | 1 | 0.002 | <0.0001 | 0.246 | 0.093 |
|  | 2 | 0.004 | 0.085 | 0.384 | 0.143 |
|  | 7 | 0.008 | 0.145 | 0.527 | 0.236 |
|  | 28 | 0.014 | 0.154 | 0.565 | 0.319 |
|  | 59 | 0.020 | 0.163 | 0.628 | 0.383 |
| Fever | 1 | 0.002 | 0.007 | 0.078 | 0.121 |
|  | 2 | 0.004 | 0.014 | 0.111 | 0.193 |
|  | 7 | 0.008 | 0.047 | 0.254 | 0.292 |
|  | 28 | 0.014 | 0.132 | 0.393 | 0.357 |
|  | 59 | 0.020 | 0.152 | 0.474 | 0.417 |
| Hypothermia | 1 | 0.002 | 0.095 | 0.483 | 0.049 |
|  | 2 | 0.004 | 0.156 | 0.653 | 0.093 |
|  | 7 | 0.008 | 0.278 | 0.785 | 0.180 |
|  | 28 | 0.014 | 0.536 | 0.923 | 0.229 |
|  | 59 | 0.020 | 0.655 | NA | 0.283 |
| No movement | 1 | 0.002 | 0.271 | 0.462 | 0.047 |
|  | 2 | 0.004 | 0.482 | 0.679 | 0.081 |
|  | 7 | 0.008 | 0.617 | 0.799 | 0.166 |
|  | 28 | 0.014 | 0.704 | 0.915 | 0.221 |
|  | 59 | 0.020 | 0.778 | 0.964 | 0.269 |
| Convulsion | 1 | 0.002 | 0.059 | 0.134 | 0.104 |
|  | 2 | 0.004 | 0.059 | 0.346 | 0.154 |
|  | 7 | 0.008 | 0.133 | 0.640 | 0.242 |
|  | 28 | 0.014 | 0.133 | 0.800 | 0.315 |
|  | 59 | 0.020 | 0.157 | 0.916 | 0.371 |
| Poor feeding | 1 | 0.002 | 0.051 | 0.298 | 0.050 |
|  | 2 | 0.004 | 0.087 | 0.495 | 0.077 |
|  | 7 | 0.008 | 0.274 | 0.682 | 0.138 |
|  | 28 | 0.014 | 0.382 | 0.809 | 0.188 |
|  | 59 | 0.020 | 0.382 | 0.893 | 0.236 |

^a^Fast breathing (=respiratory rate >60 breaths per minute), chest indrawing (=severe chest indrawing), fever

(=temperature > 38^o^C), hypothermia (=temperature <35.5 ^o^C), no movement (=no movement or movement only

on stimulation), convulsions (by maternal report), poor feeding (=not able to feed at all or stopped feeding well)

^b^Cox regression analysis was used to examine patterns of CHW identification of each sign: a single sign found

alone (only this sign), the sign found with at least one other sign (with other signs), any sign(s) found other than

the particular sign [only other sign(s)] compared to not finding any sign in association with all-cause mortality

**Table S5. Association of community health worker (CHW) identification of signs of possible serious bacterial infection (PSBI)^a^ alone or in combination with other signs of PSBI with mortality of infants 0-<7 days (216,145 CHW assessments, 62,922 infants, 867 deaths)**

| Sign^a^ | Category^b^ | CHW visits (N) | Infants (N) | Total exposure time (days) | Deaths (N) | Mortality rate/1000 child-days | Hazard ratio (HR) | | | | | | |
| --- | --- | --- | --- | --- | --- | --- | --- | --- | --- | --- | --- | --- | --- |
|  |  |  |  |  |  |  | **Unadjusted** | | | **Adjusted^c^** | | | |
|  |  |  |  |  |  |  | **HR** | **95% confidence limits** | | **HR** | **95% confidence limits** | | **P-value** |
| Fast breathing | Yes (only this sign) | 3,607 | 2,870 | 4,766 | 41 | 8.6 | 7.3 | 5.3 | 10.1 | 6.9 | 5.0 | 9.6 | <0.0001 |
|  | Yes (with other sign/s) | 1,688 | 1,565 | 1,881 | 179 | 95.1 | 71.0 | 59.3 | 85.1 | 60.9 | 50.7 | 73.2 | <0.0001 |
|  | No (but other sign/s) | 3,898 | 3,421 | 4,735 | 272 | 57.4 | 45.6 | 39.0 | 53.4 | 38.8 | 33.1 | 45.5 | <0.0001 |
|  | No (no sign) | 206,952 | 62,235 | 356,094 | 375 | 1.1 | 1.0 |  |  |  |  |  |  |
| Severe chest indrawing | Yes (only this sign) | 147 | 142 | 226 | 4 | 17.7 | 19.6 | 7.3 | 52.4 | 17.6 | 6.6 | 47.1 | <0.0001 |
|  | Yes (with other sign/s) | 658 | 618 | 788 | 83 | 105.4 | 87.8 | 69.2 | 111.5 | 74.1 | 58.2 | 94.4 | <0.0001 |
|  | No (but other sign/s) | 8,388 | 6,829 | 10,368 | 405 | 39.1 | 31.0 | 26.9 | 35.7 | 27.6 | 23.9 | 31.9 | <0.0001 |
|  | No (no sign) | 206,952 | 62,235 | 356,094 | 375 | 1.1 | 1.0 |  |  |  |  |  |  |
| Fever | Yes (only this sign) | 982 | 915 | 1,462 | 12 | 8.2 | 5.9 | 3.3 | 10.5 | 5.1 | 2.9 | 9.1 | <0.0001 |
|  | Yes (with other sign/s) | 695 | 669 | 930 | 38 | 40.8 | 30.1 | 21.5 | 42.0 | 25.4 | 18.1 | 35.6 | <0.0001 |
|  | No (but other sign/s) | 7,516 | 6,059 | 8,990 | 442 | 49.2 | 40.3 | 35.0 | 46.2 | 36.0 | 31.3 | 41.5 | <0.0001 |
|  | No (no sign) | 206,952 | 62,235 | 356,094 | 375 | 1.1 | 1.0 |  |  |  |  |  |  |
| Hypothermia | Yes (only this sign) | 729 | 654 | 716 | 33 | 46.0 | 39.2 | 27.5 | 56.0 | 30.1 | 21.0 | 43.1 | <0.0001 |
|  | Yes (with other sign/s) | 662 | 580 | 571 | 154 | 269.9 | 195.1 | 161.2 | 236.2 | 139.2 | 113.7 | 170.6 | <0.0001 |
|  | No (but other sign/s) | 7,802 | 6,370 | 10,093 | 305 | 30.2 | 24.4 | 21.0 | 28.5 | 22.7 | 19.5 | 26.5 | <0.0001 |
|  | No (no sign) | 206,952 | 62,235 | 356,094 | 375 | 1.1 | 1.0 |  |  |  |  |  |  |
| No movement | Yes (only this sign) | 88 | 82 | 110 | 12 | 109.2 | 101.3 | 57.0 | 180.0 | 83.1 | 46.6 | 148.1 | <0.0001 |
|  | Yes (with other sign/s) | 680 | 614 | 748 | 191 | 255.5 | 196.0 | 164.2 | 233.9 | 154.0 | 128.4 | 184.7 | <0.0001 |
|  | No (but other sign/s) | 8,425 | 6,852 | 10,524 | 289 | 27.5 | 22.1 | 19.0 | 25.8 | 19.9 | 17.0 | 23.2 | <0.0001 |
|  | No (no sign) | 206,952 | 62,235 | 356,094 | 375 | 1.1 | 1.0 |  |  |  |  |  |  |
| Convulsions | Yes (only this sign) | 202 | 178 | 325 | 4 | 12.3 | 11.2 | 4.2 | 30.1 | 9.7 | 3.6 | 26.0 | <0.0001 |
|  | Yes (with other sign/s) | 377 | 309 | 399 | 68 | 170.3 | 114.3 | 88.0 | 148.4 | 94.7 | 72.7 | 123.4 | <0.0001 |
|  | No (but other sign/s) | 8,654 | 7,031 | 10,657 | 420 | 39.4 | 31.8 | 27.7 | 36.6 | 28.3 | 24.5 | 32.6 | <0.0001 |
|  | No (no sign) | 206,952 | 62,235 | 356,094 | 375 | 1.1 | 1.0 |  |  |  |  |  |  |
| Poor feeding | Yes (only this sign) | 888 | 826 | 933 | 44 | 47.2 | 36.5 | 26.7 | 50.0 | 37.0 | 27.0 | 50.7 | <0.0001 |
|  | Yes (with other sign/s) | 1,326 | 1,187 | 1,350 | 258 | 190.1 | 141.3 | 120.2 | 166.2 | 115.8 | 98.0 | 136.7 | <0.0001 |
|  | No (but other sign/s) | 6,957 | 5,677 | 9,081 | 189 | 20.8 | 17.2 | 14.5 | 20.6 | 15.2 | 12.8 | 18.2 | <0.0001 |
|  | No (no sign) | 206881 | 62,223 | 356,094 | 375 | 1.1 | 1.0 |  |  |  |  |  |  |

^a^Fast breathing (=respiratory rate >60 breaths per minute), chest indrawing (=severe chest indrawing), fever (=temperature > 38^o^C), hypothermia (=temperature <35.5 ^o^C), no movement (=no movement or movement only on stimulation), convulsions (by maternal report), poor feeding (=not able to feed at all or stopped feeding well)

^b^Cox regression analysis was used to examine patterns of CHW identification of each sign: a single sign found alone (only this sign), the sign found with at least one other sign (with other signs), any sign(s) found other than the particular sign [only other sign(s)] compared to not finding any sign in association with all-cause mortality

^c^Adjusted for maternal education, place of birth, history of labor and pregnancy complications, preterm birth

**Figure S1. Kaplan-Meier probabilities of mortality for each sign of possible serious bacterial infection**

**as a binary variable over the young infant period (days 0-<60).**
